# Differential Spatiotemporal Expression of Type I and Type II Cadherins Associated With the Segmentation of the Central Nervous System and Formation of Brain Nuclei in the Developing Mouse

**DOI:** 10.3389/fnmol.2021.633719

**Published:** 2021-03-23

**Authors:** Julie Polanco, Fredy Reyes-Vigil, Sarah D. Weisberg, Ilirian Dhimitruka, Juan L. Brusés

**Affiliations:** Department of Natural Sciences, Mercy College, Dobbs Ferry, NY, United States

**Keywords:** classical cadherins, cell adhesion molecules, neural development, differential cell adhesion, tissue morphogenesis, basal ganglia, cerebral cortex, neural circuit formation

## Abstract

Type I and type II classical cadherins comprise a family of cell adhesion molecules that regulate cell sorting and tissue separation by forming specific homo and heterophilic bonds. Factors that affect cadherin-mediated cell-cell adhesion include cadherin binding affinity and expression level. This study examines the expression pattern of type I cadherins (*Cdh1*, *Cdh2*, *Cdh3*, and *Cdh4*), type II cadherins (*Cdh6*, *Cdh7*, *Cdh8*, *Cdh9*, *Cdh10*, *Cdh11*, *Cdh12*, *Cdh18*, *Cdh20*, and *Cdh24*), and the atypical cadherin 13 (*Cdh13*) during distinct morphogenetic events in the developing mouse central nervous system from embryonic day 11.5 to postnatal day 56. Cadherin mRNA expression levels obtained from *in situ* hybridization experiments carried out at the Allen Institute for Brain Science (https://alleninstitute.org/) were retrieved from the Allen Developing Mouse Brain Atlas. *Cdh2* is the most abundantly expressed type I cadherin throughout development, while *Cdh1*, *Cdh3*, and *Cdh4* are expressed at low levels. Type II cadherins show a dynamic pattern of expression that varies between neuroanatomical structures and developmental ages. Atypical *Cdh13* expression pattern correlates with *Cdh2* in abundancy and localization. Analyses of cadherin-mediated relative adhesion estimated from their expression level and binding affinity show substantial differences in adhesive properties between regions of the neural tube associated with the segmentation along the anterior–posterior axis. Differences in relative adhesion were also observed between brain nuclei in the developing subpallium (basal ganglia), suggesting that differential cell adhesion contributes to the segregation of neuronal pools. In the adult cerebral cortex, type II cadherins *Cdh6*, *Cdh8*, *Cdh10*, and *Cdh12* are abundant in intermediate layers, while *Cdh11* shows a gradated expression from the deeper layer 6 to the superficial layer 1, and *Cdh9*, *Cdh18*, and *Cdh24* are more abundant in the deeper layers. Person’s correlation analyses of cadherins mRNA expression patterns between areas and layers of the cerebral cortex and the nuclei of the subpallium show significant correlations between certain cortical areas and the basal ganglia. The study shows that differential cadherin expression and cadherin-mediated adhesion are associated with a wide range of morphogenetic events in the developing central nervous system including the organization of neurons into layers, the segregation of neurons into nuclei, and the formation of neuronal circuits.

## Introduction

During the formation of the vertebrate central nervous system (CNS), neuronal precursors sort out and organize into functionally distinct histological structures, including stratified cell layers or laminae and neuronal aggregates or nuclei. Thereafter, neurons extend neurites and build cell–cell junctions between distantly located partners leading to the establishment of neural circuits. These morphogenetic processes are based on the ability of cells to segregate and aggregate into cohesive groups through the formation of specific adhesive bonds of varying binding affinities mediated by a repertoire of cell adhesion molecules (Steinberg, 1963, 1970; Nose et al., 1988; Steinberg and Takeichi, 1994; Gumbiner, 1996; Foty and Steinberg, 2013; Sanes and Zipursky, 2020).

Classical cadherins (*Cdh*) play a leading role in tissue morphogenesis by forming high-affinity *trans*-dimers between apposed cell membranes capable of regulating adhesive interactions necessary for specific cell sorting, aggregation of cells into groups, and the formation of complex tissue architecture (Steinberg and Takeichi, 1994; Duguay et al., 2003; Foty and Steinberg, 2005; Gumbiner, 2005; Hirano and Takeichi, 2012; Honig and Shapiro, 2020). Classical cadherins are comprised of five extracellular cadherin domains (EC), a single-pass transmembrane region, and a cytoplasmic tail with conserved protein binding sites (Hatta et al., 1988; Kemler, 1992; Pokutta and Weis, 2007; Shapiro and Weis, 2009; Oda and Takeichi, 2011). Classical cadherins are grouped in type I and type II based on the structure of the binding site in EC1 (the farthest EC from the cell membrane) that governs the mode of transdimerization (Shapiro et al., 1995; Tamura et al., 1998; Nollet et al., 2000; Boggon et al., 2002; Patel et al., 2006; Harrison et al., 2010; Honig and Shapiro, 2020). Type I cadherins include cadherin 1 (*Cdh1*), cadherin 2 (*Cdh2*), cadherin 3 (*Cdh3*), cadherin 4 (*Cdh4*), and cadherin 15 (*Cdh15*). Type II cadherins include cadherin 5 (*Cdh5*), cadherin 6 (*Cdh6*), cadherin 7 (*Cdh7*), cadherin 8 (*Cdh8*), cadherin 9 (*Cdh9*), cadherin 10 (*Cdh10*), cadherin 11 (*Cdh11*), cadherin 12 (*Cdh12*), cadherin 18 (*Cdh18*), cadherin 19 (*Cdh19*), cadherin 20 (*Cdh20*), cadherin 22 (*Cdh22*), and cadherin 24 (*Cdh24*) (Nollet et al., 2000; Gul et al., 2017). Type I cadherins possess a single critical tryptophan residue in position 2 (W2) necessary for *trans*-dimer formation. In contrast, type II cadherins have two critical tryptophan residues (W2 and W4) that cause a different folding of EC1 (Tamura et al., 1998; Boggon et al., 2002; Patel et al., 2006; Harrison et al., 2010). The differently folded EC1 found in type I and type II cadherins are incompatible with the formation of heterophilic *trans*-dimers between subtypes. However, type I and type II cadherins form high-affinity heterophilic *trans*-dimers between members of the same specificity group (Shan et al., 2000; Katsamba et al., 2009; Harrison et al., 2010; Vendome et al., 2014; Honig and Shapiro, 2020). Based on the heterophilic binding affinities, type II cadherins are divided in three specificity groups that include *Cdh6*, *Cdh9*, and *Cdh10* (here called group A), *Cdh7*, *Cdh12*, *Cdh18*, *Cdh20*, and *Cdh2*2 (here called group B), and *Cdh8*, *Cdh11*, and *Cdh24* (here called group C) (Brasch et al., 2018). The atypical *Cdh13* (also known as T-cadherin) ectodomain is comprised of five ECs linked to the cell membrane by a glycosylphosphatidylinositol (GPI) moiety, and the folding of EC1 involved in homophilic binding differs from the one found in classical cadherins (Ranscht and Dours-Zimmermann, 1991; Ciatto et al., 2010).

Cadherins have been implicated in a variety of morphogenetic events during neural development including cell migration, segmentation of the neural tube, neurite outgrowth, axon targeting, and synapse formation (Redies and Takeichi, 1996; Redies, 2000; Takeichi, 2007; Hirano and Takeichi, 2012; Sanes and Zipursky, 2020). Experimental manipulation of cadherin expression *in vivo* in developing motor neurons caused inappropriate sorting and ectopic localization into neuronal pools within the ventral horn of the spinal cord (Price et al., 2002). The study provided direct evidence that combinatorial expression of classical cadherins subtypes is necessary for the sorting and aggregation of motor neurons into anatomically and functionally distinct pools. Other studies of type I and type II cadherins in the developing CNS identified combinatorial expression patterns between interconnected neurons, indicating that cadherins regulate the specificity of synaptic connectivity required for the assembly of neuronal circuits (Duan et al., 2014, 2018; Basu et al., 2017). This evidence supports a morphogenetic model regulated by the combinatorial expression of classical cadherins that specify molecular identity and differential adhesive properties to distinct neuronal groups.

The present study examines mRNA expression levels of classical type I and type II cadherins and the atypical *Cdh13* throughout the developing mouse CNS [from embryonic day (E) 11.5 to postnatal day (P) 56], with the goal of identifying morphogenetic events regulated by cadherin-mediated differential cell–cell adhesion. Cadherins mRNA expression levels in each anatomical structure were obtained from *in situ* hybridization (ISH) studies carried out at the Allen Institute for Brain Science^1^ and available through the Allen Developing Mouse Brain Atlas^2^ (Lein et al., 2007; Ng et al., 2009; Thompson et al., 2014; Chou et al., 2016). This neurodevelopmental atlas is comprised of detailed analysis of genome-wide mRNA abundance at a single-cell resolution level in each neuroanatomical structure defined by the ontological organization of the mouse CNS (Dong, 2008; Watson et al., 2011) and described in the Reference Mouse Brain Atlas^3^. The present analysis focuses on the transverse neuromeric-based segmentation of the neural tube along the anterior–posterior axis, the dorsal–ventral plate-based division of the neural tube, the nuclear organization of neurons in the subpallium, and the differential expression of classical cadherins in the layers and areas of the cerebral cortex (dorsal pallium). The study shows that neuroanatomical structures are characterized by the expression of distinct combinations of type I and type II cadherins that generate varying relative adhesion levels throughout development, suggesting that differential cell adhesion contributes to the segregation of neurons into brain nuclei, neuromeric segmentation, assembly of neurons in layers, and formation of neural circuits.

## Materials and Methods

### mRNA Expression

Datasets from ISH experiments were retrieved from the Allen Institute for Brain Science by querying the Allen Developing Mouse Brain Atlas^4^ (Lein et al., 2007). Neuroanatomical structures are organized on the basis of the prosomeric model of CNS development (Puelles and Ferran, 2012) as described in the ontological Mouse Brain Atlas (Dong, 2008; Watson et al., 2011), which is used as reference atlas (Allen Institute, 2010, 2013). Search queries were built using the RESTfull Model Access (RMA) query builder^5^ using the structure_id as anatomical identifier (Supplementary Table 1) and section_data_set_id as identifier of ISH experiments corresponding to a cadherin probe and developmental age (Ng et al., 2007, 2009). The section_data_set_id numbers corresponding to the experiments used in this study are listed in Supplementary Table 2. The retrieved data were downloaded and analyzed in Excel worksheets. Tissue section images of the experiments used in this study can be retrieved using this web address: http://developingmouse.brain-map.org/experiment/show/“section_data_set_id” by replacing “section_data_set_id” with the desired experiment number from Supplementary Table 2.

The informatics data processing pipeline of the Allen Developing Mouse Brain Atlas uses a three-dimensional model of the ontology-based reference brain atlas gridded into voxels (Ng et al., 2007; Allen Institute, 2011). Gene expression statistics (obtained from the ISH experiments) for each anatomical structure delineated in the reference atlas are computed by combining the expression values of each voxel through multiple tissue sections within the same structure volume using the structure unionize module (Lein et al., 2007; Thompson et al., 2014). The data processing provides the expression density, intensity, and energy values of each neuroanatomical structure for each image-series stored in the database, which includes the data from each pixel contained within the volume of the anatomical structure. The reference atlas of each developmental stage is annotated at the lowest ontology level available: level 5 for E11.5, E18.5, P4, and p14; level 9–10 for E13.5 and 15.5; and level 11–13 for P56.

Expression energy values express the pixel intensity signal of each probe normalized to the number of pixels in the three-dimensional anatomical structure reconstructed from multiple tissue sections [Expression energy = (sum of expressing pixel intensity/sum of expressing pixels) × (sum of expressing pixels/sum of all pixels)]. Expression energy is used to compare gene expression levels between anatomical structures. The probes used for ISH experiments to detect cadherin mRNA levels have not been calibrated against an mRNA standard; therefore, quantitative differences in expression values between cadherins are only suggestive. In contrast, relative differences in mRNA expression levels of the same cadherin between anatomical structures reflect difference in gene expression levels.

To compare cadherins mRNA expression energy detected by ISH with neuronal mRNA levels detected by single cell RNA sequencing (scRNA-Seq), the expression energy values in the six layers of the frontal, parietal, temporal, and occipital cortical areas of the P56 mouse were compared to the amount of mRNA detected by scRNA-Seq in the 8-week-old mouse neocortex. Cadherins expression energy values in each layer of each cortical area were normalized to beta-actin (*Actb*) expression level, and the average of the normalized values from the six layers and four cortical areas was calculated for each cadherin. mRNA levels detected by scRNA-Seq of each cadherin and *Actb* in the ∼8-week-old mouse neocortex (1,093,785 total cells) were obtained from the scRNA-Seq database of the Allen Institute for Brain Science^6^ (Tasic et al., 2018; Miller et al., 2020). The Allen mouse transcriptomics whole cortex and hippocampus 10X genomics 2020 dataset was used for this analysis. From the 377 cell types identified, 24 non-neuronal cells were removed. The scRNA-Seq value of each cadherin in each of the 353 neuronal groups was normalized to *Actb*, and the average of the normalized values for each cadherin was calculated (Supplementary Figure 4). Data analysis and plotting were done in Excel, and statistical analysis and Pearson’s correlations were carried out using SPSS IBM software. Figures were prepared in Adobe Illustrator and Adobe Photoshop.

### Calculation of Cadherin Relative Adhesion

To correlate mRNA expression energy values with the number of cadherin proteins expressed on the cell surface, an expression energy value of 1 was arbitrarily equated to 25,000 protein molecules based on previous reports (Duguay et al., 2003). The expression levels of cadherins were used to calculate the adhesive force between two cells, as determined by the work W (in calories) required to separate two cells, based on the binding affinity (dissociation constant K_D_) as previously described (Katsamba et al., 2009). The concentration of cadherin’s EC1 participating in cell adhesion was calculated assuming the cell being spherical with a diameter of ∼10 μm and cadherin molecules equally distributed on the surface. Based on the distance between apposed cell membranes (obtained from electron micrographs) and the length of the entire cadherin’s EC domain (obtained from their crystal structure), the thickness of the space in which EC1 resides was estimated at 12 nm (Chen et al., 2005). The equilibrium monomer/dimer concentrations upon adhesion were then estimated using cadherins dissociation constants KD (Chen et al., 2005; Katsamba et al., 2009) according to Eq. 1,

(1) $$\begin{array}{c} C_{D}⇌2C_{M}\;(dissociation\;of\;dimers\;tomonomers)\\ K_{D}=\frac{{\left(C_{M}\right)}^{2}}{C_{D}} \end{array}$$

The number of cadherin monomers in contact between two cells was estimated based on a previously described model (Chen et al., 2005). Briefly, a perfectly spherical cell is in contact with 12 other cells; therefore, approximately 8% of cadherins expressed on one cell surface are in contact with one other cell. It was arbitrarily assumed that half of the expressed cadherin molecules in contact with other cells (about 4%) participate in cell–cell homodimer adhesion. The ratio of monomer/dimer concentration is equal to the ratio monomer/dimer molecules; thus, the number of cadherin dimers N between two cells can be estimated.

The adhesive force W between two cells is calculated using Eq. 3 (Chen et al., 2005). The Gibbs free energy of adhesion for one dimer molecule Δg(i,j) was calculated from the dissociation constant values K_D,_ according to Eq. 2. The calculated adhesive force W was normalized to the amount of force generated by 25,000 molecules of *Cdh2* and is referred as relative adhesion force.

(2) $$\Delta \,g\,\left(\text{i},\text{j}\right)=-\left(\frac{\text{RT}}{\text{n}}\right)\,\text{ln}\,\left({\text{K}}_{\text{D}}\right)\,n={\text{Avogadro}}^{{}^{\prime }}\,s\,\mathrm{number}$$

(3) $$\text{W}\,\left(\text{I},\text{J}\right)={\text{N}}_{\text{dimers}}\,\text{Δg}\,\left(\text{i},\text{j}\right)$$

### Pearson’s Correlation

Pearson’s correlation coefficient analysis was used to estimate positive and negative linear relationship between cadherins expression using IBM SPSS Statistics (Field, 2013). Statistically significant Pearson’s correlation *r* coefficients are reported at *P* < 0.01 confidence interval. Pearson’s correlation analysis was performed between pairs of individual cadherins and between groups of cadherins using mRNA expression energy values obtained from the Allen Developing Mouse Brain Atlas as described above.

## Results

### Type I and Type II Cadherins Expression in the Developing Mouse CNS

The neuroanatomical organization used by the Allen Brain Reference Atlas of the developing mouse (see text footnote 3) is based on the ontogenetic relationships between anatomical structures (Puelles and Rubenstein, 2003, 2015; Dong, 2008; Allen Institute, 2013; Puelles et al., 2013). This ontology-based neuroanatomy emphasizes the progressive regionalization, stratification, and nuclei formation as structures derived from a previous anatomical structure, and therefore facilitates the analysis of the developmental regulation of gene expression and their role in the formation of the CNS (Dong, 2008; Watson et al., 2011). The ontology-based reference atlas organizes the mouse CNS in thirteen ontological levels that expand from the early segmentation of the neural tube (level 1) into forebrain, midbrain, hindbrain, and spinal cord, to the complete set of neuroanatomical structures including cell layers and nuclei observed in the adult CNS (levels 11–13), which are similar to the ones described in the stereotaxic mouse brain atlas and in classical human neuroanatomy (Parent, 1996; Franklin and Paxino, 2019) [abbreviations of neuroanatomical structures used in the Allen Reference Atlas (see text footnote 3) and followed throughout the present study can be found in Supplementary Table 1].

**FIGURE 1:**
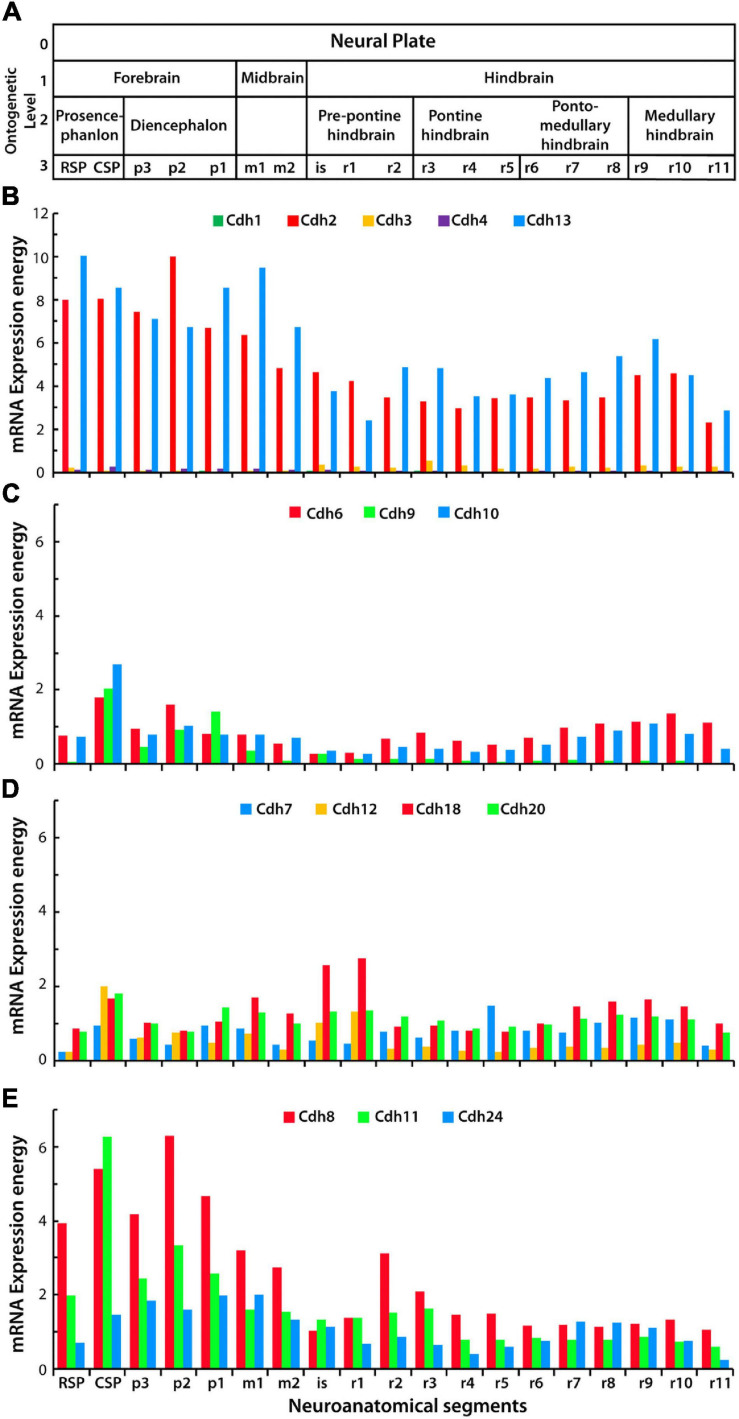
mRNA expression levels of classical cadherins and atypical *Cdh13* in the young adult (P56) mouse CNS. **(A)** Anatomical divisions of the developing CNS up to ontological level 3 based on the prosomeric model. **(B–E)** Cadherins mRNA expression energy values in each segment of the neural tube along the anterior–posterior axis (anterior is to the left) detected by ISH. **(B)** mRNA expression energy of type I cadherins *Cdh1*, *Cdh2*, *Cdh3*, *Cdh4*, and the atypical *Cdh13*. **(C)** mRNA expression energy of type II cadherins group A, *Cdh6*, *Cdh9*, and *Cdh10*. **(D)** mRNA expression energy of type II group B, *Cdh7*, *Cdh12*, *Cdh18*, and *Cdh20*. **(E)** mRNA expression energy of type II cadherins group C, *Cdh8*, *Cdh11*, and *Cdh24*. mRNA expression energy is defined as the sum of expressing pixel intensities divided by the number of pixels (see section “Materials and Methods”). RSP, rostral secondary prosencephalon; CSP, caudal secondary prosencephalon; p, prosomere; m, mesomere; is, isthmus; r, rhombomere.

This study focused on type I cadherins *Cdh1*, *Cdh2*, *Cdh3*, and *Cdh4*, type II cadherins *Cdh6*, *Cdh7*, *Cdh8*, *Cdh9*, *Cdh10*, *Cdh11*, *Cdh12*, *Cdh18*, *Cdh20*, and *Cdh24*, and the atypical *Cdh13*, which is linked to the cell membrane *via* a GPI moiety. Classical cadherins *Cdh5*, *Cdh15*, *Cdh19*, and *Cdh2*2 were excluded from this analysis because expression values were not reported in the Allen Developing Mouse Brain Atlas and/or their binding affinity has not been determined. To examine the expression levels of classical cadherins in the developing mouse CNS, the mRNA expression energy values were obtained from the Allen Developing Mouse Brain Atlas using the RMA query builder and the structure unionize module (see section “Materials and Methods”). Expression energy represents the sum of the signal intensity of all pixels within an anatomical structure normalized to the number of expressing pixels, and it is a useful unit for comparing gene expression levels between structures. Figure 1 shows the expression energy values of cadherins along the anterior–posterior axis in the young adult P56 mouse CNS. Ontological level 3 describes nineteen segments from the anterior secondary prosencephalon to the most posterior rhombomere r11 (Figure 1A). *Cdh2* and *Cdh13* are abundantly expressed throughout the CNS with similar expression levels in each segment. The forebrain shows the highest expression values, while the midbrain and hindbrain are consistently lower (Figure 1B). Type I *Cdh1*, *Cdh3*, and *Cdh4* are detected at low levels throughout the CNS. Type II cadherin group A and B are expressed at moderate levels and their expression varies along the anterior–posterior axis. *Cdh6* and *Cdh10* are uniformly expressed, while *Cdh9* is more abundant in the forebrain and progressively declines from the midbrain to the posterior end of the CNS (Figure 1C). Type II cadherins group B expression varies at each neuromeric segment with a noticeable increase of *Cdh18* in the midbrain region (Figure 1D). Type II group C *Cdh8* and *Cdh11* are expressed in a pattern similar to the one observed for *Cdh2*, with higher expression in the forebrain as compared to the midbrain and hindbrain (Figure 1E). mRNA expression level of cadherins here reported may differ from the ones reported in other studies using ISH. For instance, expression of certain classical cadherins, including *Cdh1* and *Cdh4*, has been detected in particular neural structures and are involved in developmental processes including neural tube segmentation (Matsunami and Takeichi, 1995; Redies and Takeichi, 1996; Inoue et al., 2001). Discrepancies in cadherin expression levels found between studies may be caused by methodological differences, including probes used for ISH, and by differences in anatomical classifications and in signal intensity quantification. To examine whether cadherins mRNA expression energy detected by ISH reflects neuronal mRNA levels detected by scRNA-Seq, the expression energy values in the P56 mouse isocortex were compared to the amount of mRNA detected by scRNA-Seq in the 8-week-old mouse neocortex (Tasic et al., 2018; Miller et al., 2020) (see section “Materials and Methods”). The comparison shows that *Cdh4*, *Cdh9*, *Cdh10*, and *Cdh18* expression levels may be underestimated by ISH; however, no significant statistical difference was detected between groups (Supplementary Figure 4).

**FIGURE 2:**
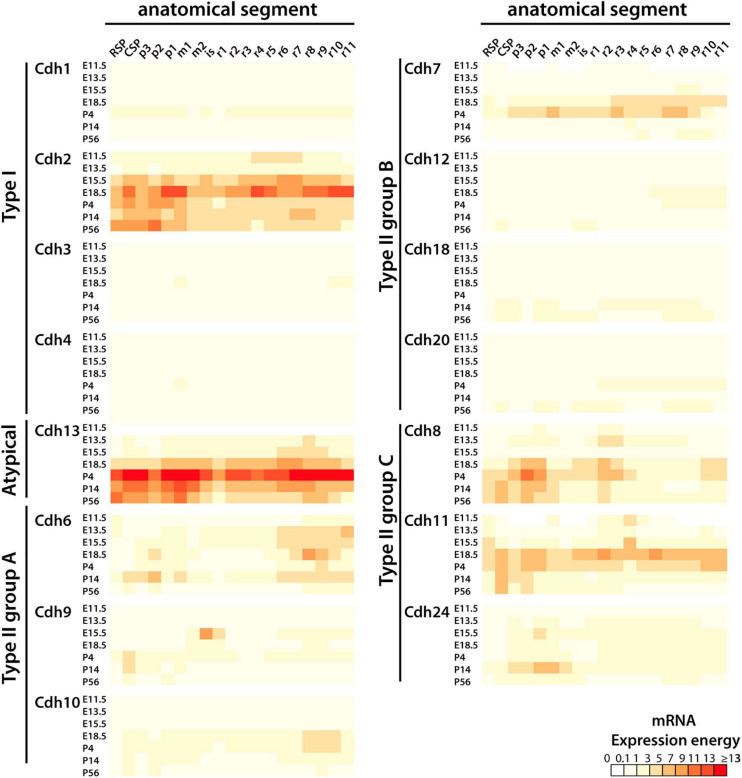
Developmental expression of classical cadherins and *Cdh13* mRNA along the anterior–posterior axis (anterior is to the left). The heatmap displays the mRNA expression energy detected by ISH of type I (*Cdh1*, *Cdh2*, *Cdh3*, and *Cdh4*) and type II cadherin group A (*Cdh6*, *Cdh9*, and *Cdh10*), group B (*Cdh7*, *Cdh12*, Cadh18, and *Cdh20*), group C (*Cdh8*, *Cdh11*, and *Cdh24*), and the atypical *Cdh13* in each neuroanatomical segment (at ontological level 3) at E11.5, E13.5, E18.5, P4, P14, and P56. RSP, rostral secondary prosencephalon; CSP, caudal secondary prosencephalon; p, prosomere; m, mesomere; is, isthmus; r, rhombomere.

The heatmap shown in Figure 2 displays the developmental pattern of cadherins mRNA expression in the mouse CNS from E11.5 to P56 (mRNA expression energy values are shown in Supplementary Table 3). All cadherins were detected in most anatomical structures throughout development; however, only a subgroup of cadherins were expressed at significant levels (a significant level of expression is considered ≥ 1 unit of expression energy). As observed at P56, cadherins expression varies along the anterior–posterior axis and between developmental stages. *Cdh2* is the only classical type I cadherin significantly expressed throughout the CNS. *Cdh1*, *Cdh3*, and *Cdh4* were detected at values below 1 unit of expression energy at all developmental stages. *Cdh2* expression peaks at E18.5 in the diencephalon, pontine hindbrain, and medullary hindbrain. *Cdh13* shows a similar expression pattern as *Cdh2*; however, *Cdh13* expression peaks at P4 instead of E18.5. *Cdh2* and *Cdh13* expression levels and localization are highly correlated (Person’s correlation at E13.5 *r* = 0.729, E15.5 *r* = 0.86, P14 *r* = 0.687, and P56 *r* = 0.763; *p* < 0.01). *Cdh6* is the most widely expressed type II group A cadherin. It is more abundant in the forebrain and in the pontomedullary and medullary hindbrains. *Cdh9* and *Cdh10* expression is restricted to some segments of the CNS and shares a similar anterior–posterior expression pattern in the caudal secondary prosencephalon and medullary hindbrain, with the exception of a distinctly high level of expression of *Cdh9* in the isthmus and rhombomere r1 at E15.5 and E18.5. This expression pattern is unique among all cadherins suggesting that *Cdh9* contributes to the formation of anatomical structures derived from the isthmus and rhombomere r1 region including the cerebellum. *Cdh7* is the most abundant type II group B cadherin and displays higher expression levels during late embryonic (E18.5) and early postnatal (P4) life. *Cdh12*, *Cdh18*, and *Cdh20* are expressed at low levels with the exception of *Cdh18* that shows higher expression in the midbrain and medullary hindbrain at P56. Type II group C *Cdh8* and *Cdh11* are the most abundant type II cadherins displaying high expression by the end of embryonic development and at postnatal ages. Their expression overlaps in the forebrain and midbrain and it is reciprocal in the pontine and pontomedullary hindbrains (rhombomeres r3–r8). Both cadherins are distinctively expressed in the diencephalon at P4, suggesting a role in the separation between the diencephalon and the midbrain. *Cdh24* is expressed at lower levels compared to *Cdh8* and *Cdh11*, and its highest expression is in the diencephalon and anterior midbrain region. This analysis shows that in general, classical cadherins expression increases from E11.5 to E18.5, peaks during late embryonic (E18.5) and early postnatal (P4) life, and declines in the young adult (P56). During embryonic development, cadherins expression levels are higher in the hindbrain and shift to the forebrain during postnatal life. Their expression varies along the anterior–posterior axis indicating that each neuromeric segment expresses a distinct and developmentally regulated combination of cadherins.

### Differential Cadherin-Mediated Adhesion Associated With the Segmentation of the Neural Tube Along the Anterior–Posterior Axis

The neural tube develops along anterior–posterior and dorsal–ventral axes (Martinez et al., 2012). Transverse tissue boundaries that extend from the roof to the floor plate create tissue segments (also known as neuromeres) along the anterior–posterior axis, while divisions along the dorsal–ventral axis create plate-based longitudinal separations of the neural tissue (Puelles and Rubenstein, 2003; Watson et al., 2011). Neuromeric segments (described at ontogenetic level 3) represent neural precursor domains that generate nuclear and laminar tissue structures observed in the adult brain. Cadherin-mediated adhesion, as determined by the work (W) required to separate two cells, depends on their binding affinity and number of *trans*-cadherin dimers formed between the two apposed cells, and is directly related to the number of molecules on the cell surface (Duguay et al., 2003; Foty and Steinberg, 2005; Katsamba et al., 2009; Vendome et al., 2014; Brasch et al., 2018). To estimate the effect that changes in cadherin expression have on the adhesive properties of each segment of the CNS, the relative adhesion of each cadherin was calculated based on their homophilic binding affinity and mRNA expression level (determined by ISH). This analysis assumed that differences in mRNA expression levels are directly proportional to differences in protein expression levels. To correlate mRNA expression with the number of cadherin molecules expressed, an mRNA expression energy value of 1 was considered to represent 25,000 molecules of cadherin. The level of expression of each cadherin in each anatomical structure and their binding affinities were used to calculate relative adhesive forces (W in calories) as described in Section “Materials and Methods” (Katsamba et al., 2009; Vendome et al., 2014; Brasch et al., 2018).

**FIGURE 3:**
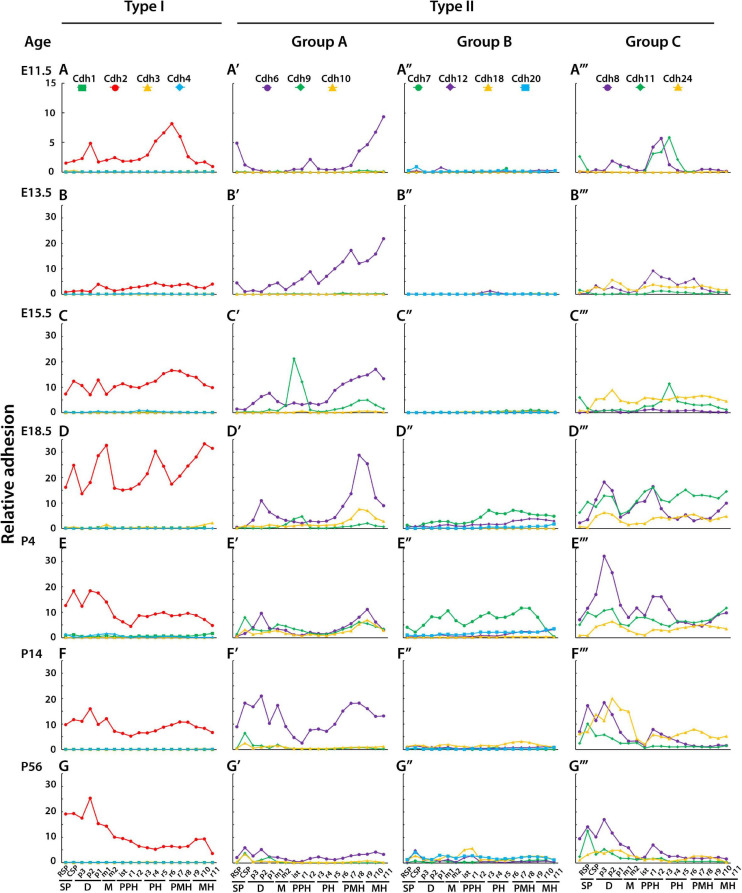
Relative adhesion values of classical cadherins during CNS development. The relative adhesion of cadherins in each neuroanatomical segment (at ontogenetic level 3) along the anterior–posterior axis was estimated based on the level of cadherin mRNA expression (detected by ISH) and homophilic binding affinity (anterior is to the left). One unit of mRNA expression energy was normalized to 25,000 cadherin molecules, and adhesion forces were estimated based on the number of molecules and homophilic binding affinity as previously described (see section “Materials and Methods”). **(A–G)** type I cadherins; **(A′–G′)** type II cadherins group A; **(A′′–G′′)** type II cadherins group B; **(A′′′–G′′′)** type II cadherins group C. **(A–A′′′)** E11.5; **(B–B′′′)** E13.5; **(C–C′′′)** E15.5; **(D–D′′′)** E18.5; **(E–E′′′)** P4; **(F–F′′′)** P14; and **(G–G′′′)** P56. SP, secondary prosencephalon; D, diencephalon; M, midbrain; PPH, prepontine hindbrain; PH, pontine hindbrain; PMH, pontomedullary hindbrain; MH, medullary hindbrain; RSP, rostral secondary prosencephalon; CSP, caudal secondary prosencephalon; p, prosomere; m, mesomere; is, isthmus; r, rhombomere.

Figure 3 shows the relative adhesion of classical cadherins along the anterior–posterior axis of the CNS during embryonic and postnatal development. At E11.5, seven regions can be identified by different relative adhesion levels of *Cdh2*, *Cdh6*, *Cdh8*, and *Cdh11* (Figures 3A–A′′′). From anterior to posterior, high levels of *Cdh6* relative adhesion are observed in the secondary prosencephalon, while higher *Cdh2* adhesion is observed in the diencephalon. Differences in *Cdh8* and *Cdh11* adhesion are detected between the midbrain and prepontine regions followed by a decline in *Cdh8* and *Cdh11* and an increase in *Cdh2* adhesion between pontine and pontomedullary segments. The most posterior medullary region shows high levels of *Cdh6* adhesion (see Supplementary Figure 1 for representative images of ISH experiments). At 13.5, *Cdh2* relative adhesion has decreased and flattened along the anterior–posterior axis, while *Cdh6* relative adhesion is higher in the midbrain, pontine, and medullary segments. *Cdh8* shows a similar pattern of relative adhesion as the one observed at E11.5, with a noticeable higher value in the pontine rhombomeres (r2–r5). *Cdh11* relative adhesion has decreased and flattened along the axis, and *Cdh24* adhesion has increased in the midbrain region (Figures 3B–B′′′). At E15.5, *Cdh9* relative adhesion is higher in the isthmus and prepontine segments, while type II group C cadherins adhesion remains higher in the pontine rhombomeres r3 to r5 (Figures 3C–C′′′). By the end of embryonic development (E18.5), the relative adhesion of *Cdh2* and *Cdh6* has increased substantially (Figures 3D–D′′′). *Cdh2* adhesion appears higher in the prosencephalon, diencephalon, pontine, and posterior rhombomeres, while *Cdh6* adhesion remains high in the posterior medullary region. No significant changes in group C cadherins relative adhesion are observed at this stage. From birth to young adult, *Cdh2* adhesion remains prominent in the anterior regions of the CNS (prosencephalon, diencephalon, and midbrain), *Cdh6* relative adhesion is higher in the prosencephalon and prepontine hindbrain and type II group C cadherins relative adhesion is higher toward the anterior regions of the CNS. (Figures 3E–E′′′,F–F′′′,G–G′′′ correspond to P4, P14, and P56, respectively). Low levels of relative adhesion are observed for type I (*Cdh1*, *Cdh3*, and *Cdh4*) and type II group B cadherins with the exception of *Cdh7* that shows intermediate values of adhesion after birth (Figure 3E′′).

Although no precise boundaries between segments of the neural tube may be drawn from this analysis, the results show differential cadherin adhesion between distinct segments and regions of the neural tube. From anterior to posterior, differences in relative adhesion between segments are as follows: r1 and r2 are associated with an increase in *Cdh6*, *Cdh8*, and *Cdh11* adhesion; r2 and r3 are associated with a decrease in *Cdh6* and an increase in *Cdh8* adhesion; r3 and r4 correspond with an increase in *Cdh11* and *Cdh2* adhesion and a decrease in *Cdh8*; r4 and r5 are associated with an increase in *Cdh2* and a decrease in *Cdh11* adhesion; and r7 and r8 are associated with a decrease in *Cdh2* and an increase in *Cdh6* adhesion. The complementary and reciprocal patterns of *Cdh2* and type II cadherins group A and C relative adhesion associated with different regions of the neural tube suggest that combinatorial cadherin-mediated adhesion contributes to the compartmentalization of the neural tube along the anterior–posterior axis.

**FIGURE 4:**
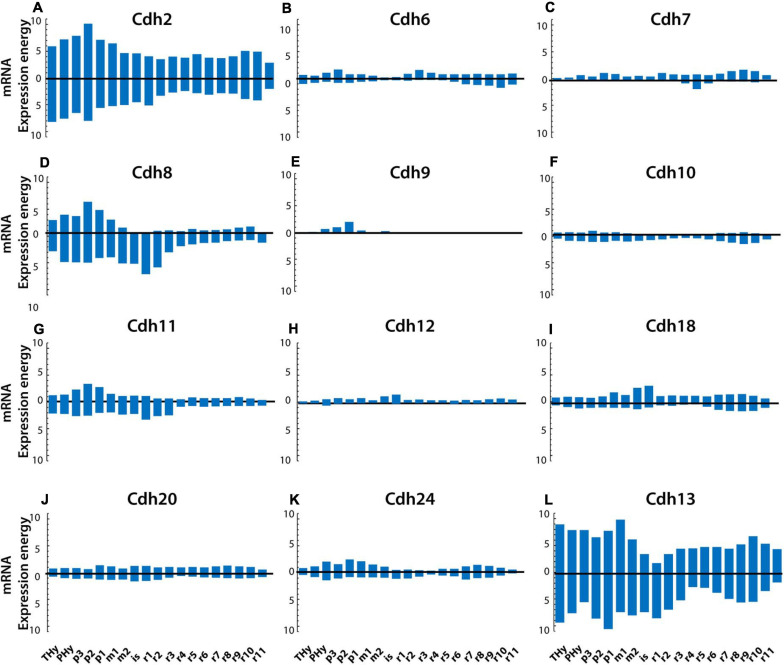
Cadherin mRNA expression levels in the alar and basal plates in the young adult mouse (P56) CNS. Panels show the mRNA expression energy of cadherins in each neuroanatomical segment along the anterior–posterior axis (anterior is to the left) detected by ISH. Values above the horizontal zero line show mRNA expression in the alar plate, and values below the zero line show mRNA expression levels in the basal plate. **(A)** *Cdh2*; **(B)** *Cdh6*; **(C)** *Cdh7*; **(D)** *Cdh8*; **(E)** *Cdh9*; **(F)** *Cdh10*; **(G)** *Cdh11*; **(H)** *Cdh12*; **(I)** *Cdh18*; **(J)** *Cdh20*; **(K)** *Cdh24*; and **(L)** *Cdh13*. THy, terminal (rostral) hypothalamus; PHy, peduncular hypothalamus; p, prosomere; m, mesomere; is, isthmus; r, rhombomere.

The plate-based partitions divide the neural tube into roof, alar, basal, and floor plates (described at ontological level 5). Figure 4 shows the expression levels of classical cadherins at P56 in the alar and basal plates in each segment along the anterior–posterior axis. The boundary between the alar and basal plates is represented by the horizontal zero line, the bars above the zero line show cadherin expression energy in the alar plate, while the bars below the zero line display the expression energy in the basal plate. *Cdh2* and *Cdh13* are the most abundantly expressed cadherins in both the alar and basal plates, followed by *Cdh8* and *Cdh11* (Figures 4A,L,D,G respectively). In most segments, cadherin expression levels in the alar and basal plates are mirror images along the anterior–posterior axis, with some deviations observed in *Cdh8* and *Cdh13* showing higher expression in the basal plate of anterior rhombomeres (Figures 4D,L).

To examine whether cadherin expression levels differ between the alar and basal plate in each CNS segment throughout development, the ratios of expression energy between the two plates along the anterior–posterior axis were calculated at each developmental stage (Supplementary Figure 2). The values on the horizontal zero line indicate a ratio of 1 (equal expression energy in the alar and basal plates), values above the zero line indicate higher expression in the alar plate, while values below the zero line indicate higher expression in the basal plate. Regional variations in the amount of cadherins expressed between plates are observed at varying developmental stages along the anterior–posterior axis. *Cdh2* is equally expressed at all ages except for a higher expression at E11.5 in the diencephalic and pontomedullary regions (Supplementary Figures 2A–G). The atypical *Cdh13* shows a large increase in the basal plate in the medullary hindbrain at E11.5 (Supplementary Figure 2A) and up to fourfold higher expression in the basal plate in the anterior rhombomeres r2 and r3 during postnatal development (Supplementary Figures 2E–G). Type II group A cadherins are also expressed at similar levels in both plates except for an increase in *Cdh6* in the midbrain and prepontine hindbrain (Supplementary Figure 2B′) and a sharp increase of *Cdh9* in the alar plate at E15.5 (Supplementary Figure 2C′). *Cdh20* shows a narrow increase in the basal plate of the prosencephalon at E11.5 (Supplementary Figure 2A′). Thereafter, no substantial differences between plates are observed in all type II group B cadherins. The largest fluctuations in the expression of cadherins between alar and basal plates are observed in type II group C cadherins. At E11.5, *Cdh8* and *Cdh11* show an increase in the alar plate in the prosencephalon and anterior rhombomeres (Supplementary Figure 2A′′′). Thereafter, both cadherins are expressed at higher levels in the basal plate with the largest difference observed at E13.5, P14, and P56 (Supplementary Figures 2B′′′,G′′′,F′′′ respectively). These results show that most cadherins are expressed at similar levels in the alar and basal plates along the anterior–posterior axis throughout development with regional differences in restricted areas and at particular ages. Differences between plates are observed in the midbrain and pontine hindbrain that coincide with the expansion of the alar plate that generate the mesencephalic tectum and the cerebellum. The small differences in cadherin expression levels between alar and basal plates along the anterior–posterior axis suggest that differential cadherin-mediated adhesion plays a role in the formation of boundaries at particular segments of the neural tube but not along the entire anterior–posterior axis.

### Type I and Type II Cadherins Associated With the Nuclear Organization of the Subpallium

The alar plate of the evaginated telencephalic vesicle is divided into pallium (that includes the cerebral cortex) and subpallium (that includes the basal subdivision of the telencephalon and the basal ganglia) (Dong, 2008; Watson et al., 2011). The portion of the neural tube that gives rise to the subpallium is divided into a proliferative thin layer or ventricular zone adjacent to the inner ventricles, and a thicker mantel zone subdivided in periventricular, intermediate, and superficial strata (Dong, 2008). The subpallial neural tube is comprised of four topological divisions, namely, the subpallial septum, the paraseptal subpallium, the central subpallium or classic basal ganglia (that includes the corpus striatum and globus pallidus), and the subpallial amygdala. These four divisions are described at ontological level 7 (in the Allen Developing Mouse Brain Atlas), and each of them is subdivided into a diagonal, pallidal, and striatal part (with the exception of the subpallial amygdala that contains a hypothalamic part). At E13.5 and E15.5, the ventricular and mantle zones are described at ontological level 9, while the periventricular, intermediate and superficial strata of the mantle zone are described at ontological level 10. During embryonic development, each of the main divisions of the subpallium is further parcellated into smaller progenitor domains characterized by the expression of different combinations of regulatory proteins that generate distinct cell groups (Medina and Abellan, 2012). In contrast to the dorsal pallium that forms the cerebral cortex with a layered organization, the three strata of the mantle zone give rise to cellular aggregates or brain nuclei including the classical basal ganglia identified in the adult mouse brain and described at level 11 of the ontogenetic atlas.

**FIGURE 5:**
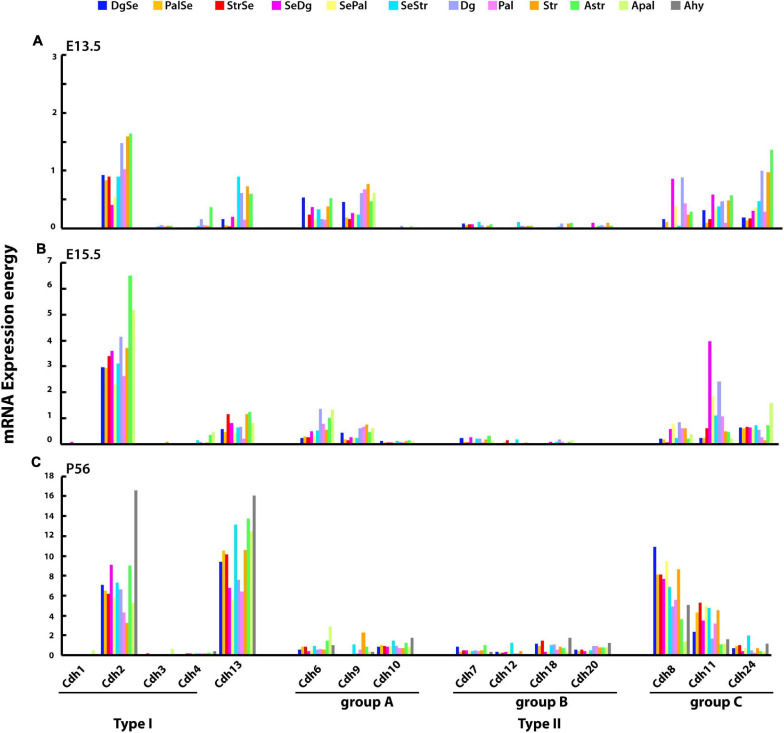
mRNA expression energy values of type I and type II cadherins and atypical *Cdh13* in the developing subpallium. Bars display the mRNA expression energy of a cadherin in each neuroanatomical segment of the developing subpallium at ontological level 8 detected by ISH. **(A)** E13.5; **(B)** E15.5; and **(C)** P56. DgSe, diagonal part of septum; PalSe, pallidal septum; StrSe, striatal septum; SeDg, septodiagonal transition area; SePal, septopallidal transition area; SeStr, septostriatal transition area (accumbens); Dg, diagonal domain; Pal, pallidum (globus pallidus complex); Str, striatum (corpus striatum); AStr, striatal amygdala; APal, pallidal amygdala; Ahy, hypothalamic amygdala.

The expression of type I and type II cadherins mRNA in the developing mouse subpallium was examined at E13.5, E15.5, and P56 in the diagonal, pallidal, and striatal parts of each of the four subpallial anatomical divisions, which are described at ontological level 8 (mRNA expression energy values at ontology levels 8–12 have been only annotated in the E13.5, E15.5, and P56 mouse brains). At E13.5, cadherins expression is relatively low with substantial differences between subpallial structures (Figure 5A). *Cdh2* is the most abundant cadherin with the highest expression observed in the diagonal, striatal, and pallidal parts of the central subpallium. Type II group A *Cdh6* and *Cdh9* are expressed at moderate levels and show varying degrees of expression between structures. Type II group B cadherins are almost undetected at this stage, while group C cadherins are expressed at moderate levels. As observed in other CNS structures, *Cdh13* correlates with the expression of *Cdh2* (Pearson’s correlation coefficient: E13.5 *r* = 0.601, E15.5 *r* = 0.677, and P56 *r* = 0.564). By E15.5, cadherins expression levels have increased two to five times without substantial changes in their expression patterns (Figure 5B). The highest expression values of *Cdh2* are observed in the central subpallium, while *Cdh11* shows a substantial increase in the paraseptal subpallium. By P56, type I and type II cadherins and *Cdh13* expression has increased significantly as compared to E15.5 (Figure 5C). *Cdh2* expression level is higher in parts of the subpallial amygdala and paraseptal subpallium. Among type II cadherins, *Cdh8* and *Cdh11* are most abundantly expressed in anatomical structures with low *Cdh2* expression.

**FIGURE 6:**
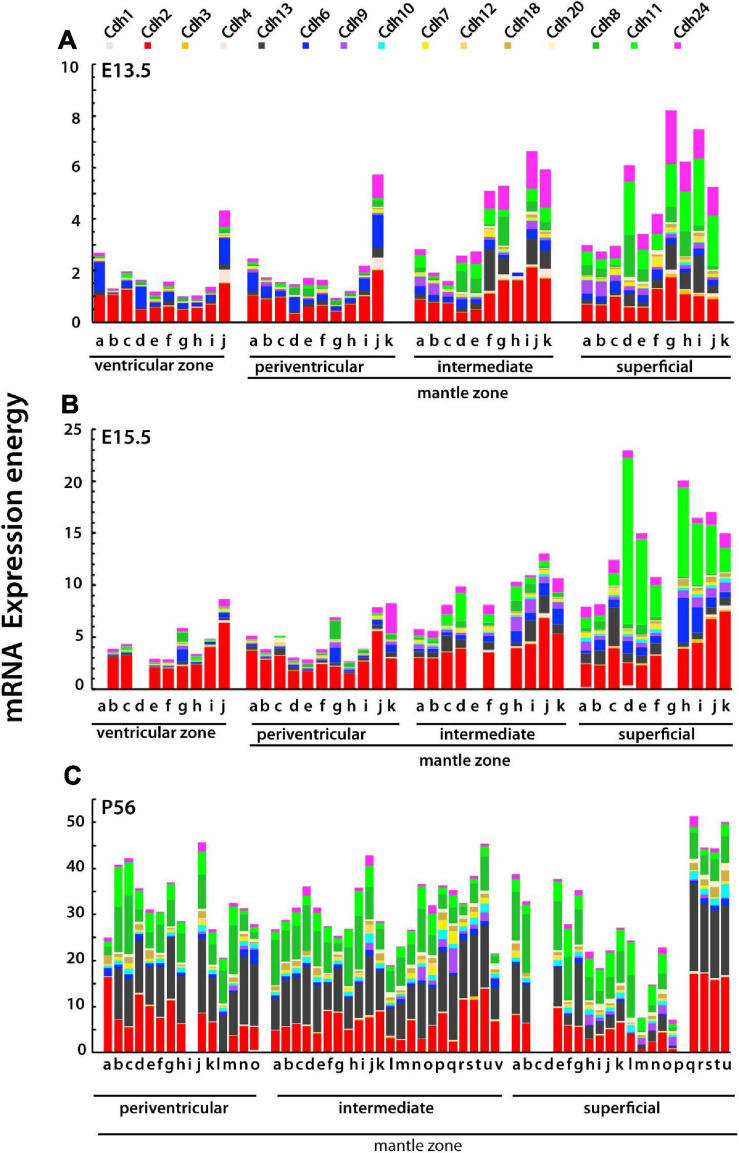
Type I and type II cadherins mRNA expression energy values in each neuroanatomical structure of the subpallium grouped by stratum at E13.5, E15.5, and P56. Each color-coded portion of the stacked bars displays the mRNA expression energy of a cadherin in each neuroanatomical structure from by ISH experiments. **(A)** E13.5 (ontological levels 9 and 10); **(B)** E15.5 (ontological levels 9 and 10); and **(C)** P56 (ontological levels 11 and 12). **(A,B)** Ventricular zone: a, DgSev; b, PalSev; c, StrSev; d, SeDgv; e, SePalv; f, SeStrv; g, Dgv; h, Palv; i, Strv; j, AStrv. Periventricular stratum: a, DgSep; b, PalSep; c, StrSep; d, SeDgp; e, SePalp; f, SeStrp; g, Dgp; h, Palp; i, Strp; j, AStrp; k, APalp. Intermediate stratum: a, DgSei; b, PalSei; c, StrSei; d, SeDgi; e, SePali; f, SeStri; g, Dgi; h, Pali; i, Stri; j, AStri; k, APali. Superficial stratum: a, DgSes; b, PalSes; c, StrSes; d, SeDgs; e, SePals; f, SeStrs; g, Dgs; h, Pals; i, Strs; j, AStrs; k, APals. **(C)** Periventricular stratum: a, MnSC; b, LSIp; c, LSD; d, TS; e, SFi; f, BSTMS; g, BSTMv; h, BSTMa; i, SePalp; j, AcbCo; k, BSTMC; l, BSTLC; m, Cau; n, ASt; o, BSTLA. Intermediate stratum: a, LSIV; b, SHy; c, LSII; d, Ld; e, LSID; f, SIBT; g, BAC; h, SePalCo; i, SePalSh; j, AcbSh; k, SIB; l, IPal; m, EPal; n, VPal; o, Put; p, VStr; q, IPAC; r, LSS; s, CeL; t, CeC; u, IA; v, CeM. Superficial stratum: a, VDB; b, MS; c, PalSes; d, StrSes; e, HDBT; f, TuSePal; g, ICjM; h, TuSeStr; i, TuPal1; j, TuPal2; k, TuPal3; l, ICjPal; m, TuStr1; n, TuStr2; o, TuStr3; p, ICjStr; q, AStrs; r, MePD; s, MePV; t, MeAD; u, MeAV. The complete names of neuroanatomical structures are listed in Supplementary Table 1.

Figure 6 displays cadherin mRNA expression in each neuroanatomical structure in the strata of the developing subpallium. At E13.5, cadherins expression increases from the ventricular zone to the superficial stratum in most subpallial structures. *Cdh2* and *Cdh6* are prominent in the ventricular and periventricular zones, *Cdh2*, *Cdh13*, and *Cdh24* are most abundant in the intermediate stratum, while *Cdh11* and *Cdh24* show higher expression levels in the superficial stratum. At E15.5, cadherins expression levels have increased substantially with overall higher values in the superficial stratum as compared to the ventricular and periventricular strata (Figure 6B). By P56, the expression of classical cadherins has increased across the three strata and displays large variability between neuroanatomical structures (Figure 6C). The most abundantly expressed cadherins are *Cdh2*, *Cdh13*, *Cdh8*, and *Cdh11*. This analysis shows that during embryonic development, each stratum of the mantle zone has a distinct classical cadherin expression profile characterized by a progressive increase toward the superficial stratum, which is primarily due to an increase in the expression of type II cadherins group C. By P56, the landscape of cadherin expression differs from the one observed during embryonic development in that most subpallial nuclei express the same profile of classical cadherins at different levels. The relative expression level of each cadherin differs substantially among structures derived from each stratum of the mantle zone, suggesting that in the adult basal ganglia differences in adhesion between nuclei are caused by varying expression levels of cadherins rather than by differences in cadherins subtypes.

To examine whether variations in the adhesive properties of different cell groups in the central subpallium are associated with the formation of the basal ganglia, the relative adhesion values of the most abundantly expressed cadherins (*Cdh2*, cadherin group A and group C) were calculated in each nucleus of the corpus striatum (striatum) and the globus pallidus complex (pallidum), which are anatomically adjacent in the adult brain. The striatum is comprised of seven nuclei that originate from different strata: the caudate originates from the periventricular stratum; the putamen, interstitial nucleus of the posterior limb of the anterior commissure and the laterostriatal stripe originate from the intermediate stratum; and the striatal part of the olfactory tuberculum and the striatal islands of Calleja originate from the superficial stratum. The globus pallidus complex includes five nuclei that originate from different strata: the lateral division of the bed nucleus of the stria terminalis derives from the periventricular stratum; the external and ventral globus pallidi derive from the intermediate stratum; and the pallidal parts of the olfactory tuberculum and the islands of Calleja originate from the superficial stratum.

**FIGURE 7:**
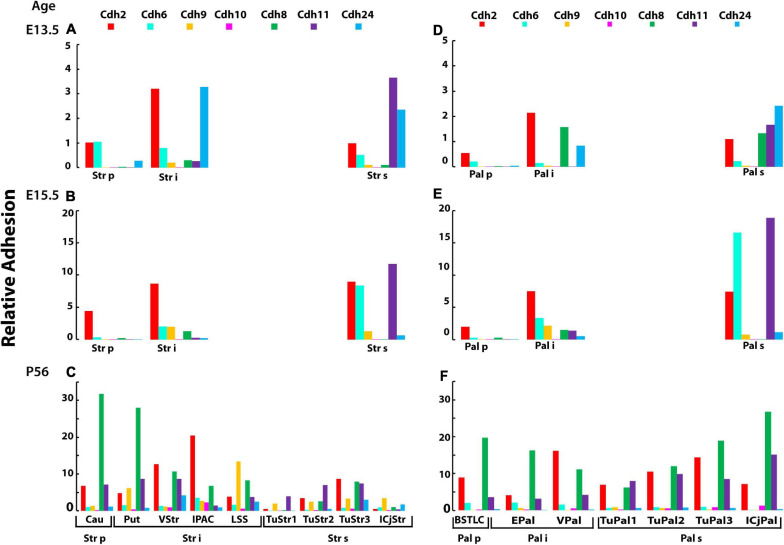
Developmental changes in cadherin mediated-relative adhesion associated with the formation of brain nuclei in the central subpallium (corpus striatum and globus pallidus). The relative adhesion values of *Cdh2*, cadherin type II group A (*Cdh6*, *Cdh9*, and *Cdh10*) and group C (*Cdh8*, *Cdh11*, and *cdh24*) were calculated based on mRNA expression level (detected by ISH) and binding affinity (see section “Materials and Methods”) in the brain nuclei derived from the mantle zone of the striatum and pallidum. **(A,D)** Relative adhesion at E13.5 in the three strata of the striatal and pallidal subdivisions, respectively; **(B,E)** relative adhesion at E15.5 in the striatal and pallidal subdivisions, respectively; and **(C,F)** relative adhesion at P56 in the nuclei derived from the three strata in the striatum and globus pallidus, respectively. Str, striatum; Pal, globus pallidal complex; p, periventricular stratum; i, intermediate stratum; s, superficial stratum; Cau, caudate nucleus; Put, putamen, VStr, ventral striatum; IPAC, interstitial nucleus of the posterior limb of the anterior commissure; LSS, laterostriatal stripe; TuStr, striatal part of olfactory tuberculum; ICjStr, striatal islands of Calleja; BSTLC, bed nucleus of the stria terminalis laterocentral division; EPal, external globus pallidum; VPal, ventral pallidum; TuPal, pallidal part of olfactory tuberculum; ICjPal, pallidal islands of Calleja.

At E13.5, the three strata of the striatum differ in their levels of relative adhesion. The periventricular stratum displays low levels of *Cdh2* and *Cdh6* adhesion, the intermediate stratum has ∼3 times higher levels of *Cdh2* and *Cdh24*, while in the superficial stratum, the relative adhesion of *Cdh11* and *Cdh24* is the most prominent (Figure 7A). This suggests that *Cdh2* and *Cdh24* may differentiate the intermediate from the periventricular strata, while *Cdh11* delineates the superficial and intermediate strata. The adjacent globus pallidus shows a pattern of relative adhesion similar to the one observed in the striatum, with the difference that *Cdh8* adhesion is substantially higher in the intermediate and superficial strata as compared to the same strata of the striatum (Figure 7D). At E15.5, *Cdh2* adhesion is similar between the intermediate and superficial strata of the striatum, while the superficial stratum displays a substantially higher adhesion values mediated by *Cdh6* and *Cdh11* (Figure 7B). The relative adhesion pattern of the striatum and the globus pallidus displays high *Cdh11* adhesion in the superficial stratum (Figure 7E).

Between E15.5 and P56, the three strata of the striatum and the globus pallidum fragment into the twelve nuclei mentioned above and display varying levels of relative adhesion (Figures 7C,F). The caudate and putamen nuclei that derive from the periventricular and intermediate strata of the striatum, respectively, show a ∼10 times increase in *Cdh8* adhesion, while each of the other nuclei of the striatum displays different profiles of adhesion (Figure 7C). *Cdh8* relative adhesion is highest in the caudate and putamen and gradually decreases toward the nuclei derived from the superficial stratum. In contrast, *Cdh2* relative adhesion is low in the caudate and increases toward the intermediate stratum up to the pallidal Island of Calleja. The striatal part of the olfactory tuberculum that derives from the superficial stratum displays lower levels of relative adhesion; however, higher values are observed in the superficial layer. *Cdh8* is most prominent in the ventral division of the BSTC nucleus (that originates from the periventricular stratum) and decreases toward the nuclei originated from the intermediate stratum (Figures 7C,F). *Cdh8* relative adhesion increases substantially toward the superficial stratum (pallidal part of the olfactory tuberculum) and reaches the highest level in the Island of Calleja. *Cdh2* and *Cdh11* relative adhesion also increases progressively toward the superficial layer in the globus pallidus (Figure 7F). This pattern is the reverse to the one observed in the striatum, in which the highest values are detected in the caudate and putamen and the lowest values are detected in the nuclei from the superficial striatum. These results suggest that differential expression of *Cdh2* and *Cdh24* segregates cells from the caudate and the putamen during embryonic development, but the difference disappears in the adult in which *Cdh8* predominates in both nuclei. The segregation of nuclei derived from the superficial stratum from the ones derived from the intermediate stratum during embryonic development appears to be driven by a higher *Cdh11* adhesion in the striatum and the globus; however, this difference also disappears in the adult brain. Despite the differential adhesive properties observed between strata during embryonic development, no clear differences are observed between the striatum and the globus pallidus. In contrast, these two nuclear complexes differ significantly in the adult brain. At P56, most of the nuclei in the central subpallium have distinct adhesive properties generated by both, distinct expression of cadherin subtypes and different expression levels. The adhesion profile of each cell group observed in the P56 brain does not always correlate with the profile observed in the embryo, suggesting that developmental regulation of cadherins expression in each cell group supports the segregation into distinct neuronal pools.

### Classical Cadherin Expression in the Dorsal Pallium (Isocortex) in the Young Adult CNS

**FIGURE 8:**
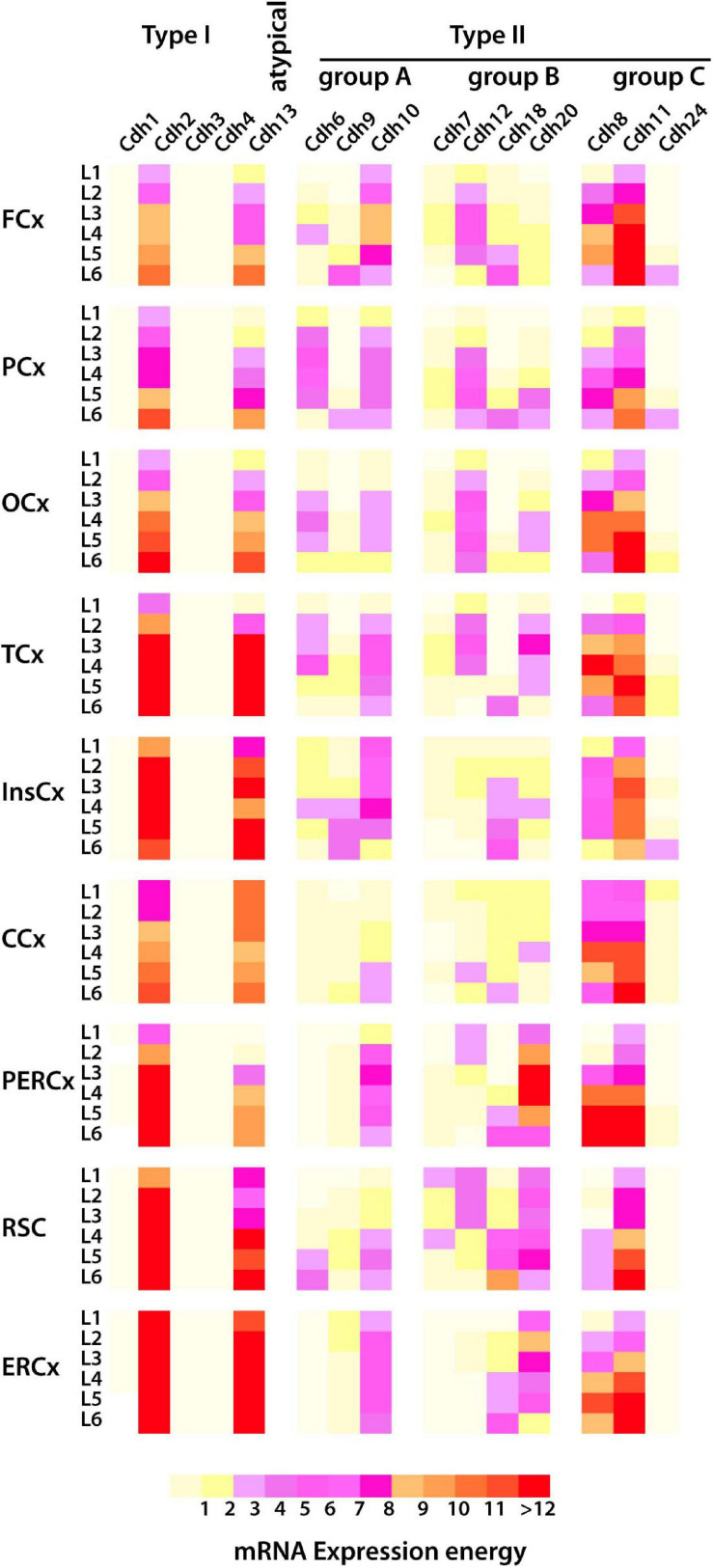
mRNA expression of type I and type II cadherins and the atypical *Cdh13* in the cortical layers of the dorsal pallium (isocortex) in the young adult mouse (P56) from ISH experiments. The heatmap displays cadherins mRNA expression energy in each layer of the nine cortical areas detected by ISH. The superficial layer 1 (L1) is at the top, and the deeper layer 6 (L6) is at the bottom. FCx, frontal cortex; PCx, parietal cortex, OCx, occipital cortex; TCx, temporal cortex; InsCx, insular cortex; CCx, cingulate cortex; PERCx, perirhinal–ectorhinal cortex; RSCx, retrosplenial cortex; ERCx, entorhinal cortex.

The mouse isocortex is divided into six layers and nine areas which include frontal, parietal, occipital, temporal, insular, cingulate, perirhinal–ectorhinal, retrosplenial, and entorhinal. Analysis of cadherin mRNA expression in the dorsal pallium at P56 shows a checkered profile that varies between cortical areas and cell layers (Figure 8) (expression values are shown in Supplementary Table 5 and representative images of ISH tissue sections are shown in Supplementary Figure 3). *Cdh2* and *Cdh13* are highly expressed in all cortical areas in a decreasing gradient from the deeper layer 6 toward the superficial layer 1. *Cdh10* is the most abundantly expressed type II cadherin group A in all cortical areas with the highest expression in the intermediate layers 2–5. *Cdh6* is also detected in the intermediate cell layers, but it is restricted to the frontal, parietal, temporal, and occipital areas, with the exception of the retrosplenial cortex in which *Cdh6* is abundant in layer 6. *Cdh9* expression is higher in layer 6 in most of the cortical areas and in layer 1 in the entorhinal cortex. Type II group B cadherins are expressed in all cortical areas and layers at varying levels. *Cdh12* is expressed in the intermediate layers of the frontal, parietal, temporal, occipital, and retrosplenial cortex. *Cdh20* is also abundant in the intermediate layers, but in contrast to *Cdh12*, it is expressed at higher levels in posterior areas of the cortex (perirhinal–ectorhinal, retrosplenial, and entorhinal cortex). *Cdh18* is expressed in the deep layers 5 and 6 of all cortical areas. Type II group C *Cdh8* expression is higher in the intermediate layers 5–3 in most cortical areas, while *Cdh11* is expressed in a decreasing gradient from layer 6 to layer 1. *Cdh24* expression is lower than *Cdh8* and *Cdh11* and it is most prominent in layers 1 and 6.

The analysis of cadherin expression in the isocortex shows a varied expression pattern in each cortical layer. *Cdh2* and *Cdh13* expression is higher in posterior areas of the cortex (peri-ectorhinal, retrosplenial, and entorhinal) that are folded under the anterior areas of the dorsal pallium. High cadherin expression levels are also detected in layers of the temporal and cingulate areas; however, no obvious pattern of cadherin expression along the anterior–posterior orientation and across cortical layers is observed. *Cdh2*, *Cdh8*, *Cdh11*, and *Cdh13* are the most abundantly expressed cadherins across the dorsal pallium, while the other type II cadherins are expressed at varying levels in different cortical areas. Therefore, the repertoire of cadherin expression appears to be distinct in each cortical area and cell layer. This expression pattern supports the notion that combinatorial expression of type I and type II cadherins provides a molecular identity to each area and layer of the cortex that contribute to the positioning of the neurons and to the formation of specific neuronal contacts with cortical and subcortical neuroanatomical structures (Inoue and Sanes, 1997; Kadowaki et al., 2007; Krishna et al., 2009, Krishna et al., 2011).

### Correlation of Cadherin Expression Between the Isocortex and the Subpallium

**FIGURE 9:**
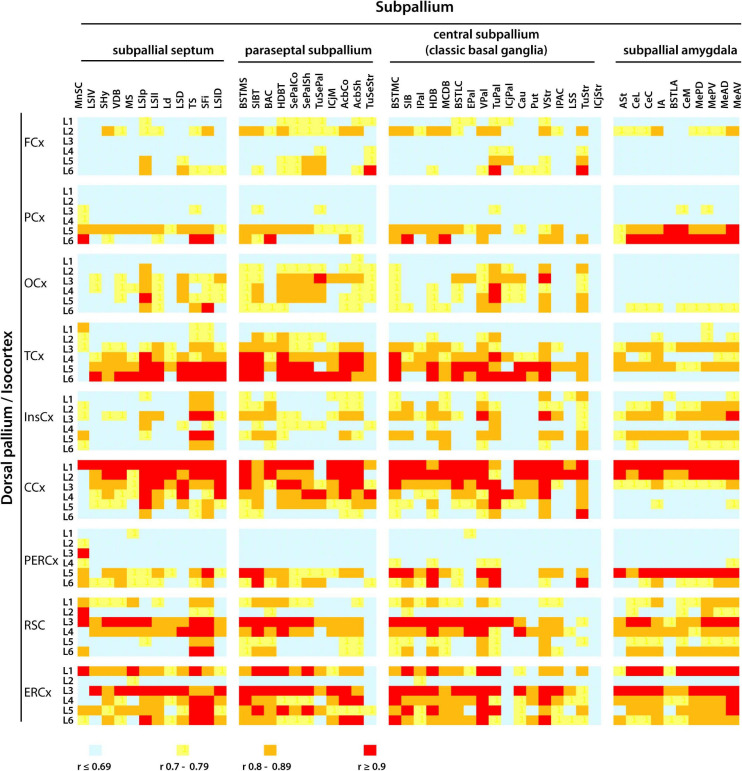
Pearson’s correlation analysis of cadherins mRNA expression pattern between cortical areas and the nuclei of the subpallium in the P56 mouse CNS. The pattern of mRNA expression (detected by ISH) of *Cdh2*, *Cdh13*, type II cadherins group A (*Cdh6*, Ch9, and *Cdh10*), group B (*Cdh7*, *Cdh12*, *Cdh18*, and *Cdh24*), and group C (*Cdh8*, *Cdh11*, and *Cdh24*) in layers (L1–L6) of each cortical area was correlated with the cadherin expression pattern in each of the fifty neuroanatomical divisions of the subpallium described at ontological level 11 and 12. The colors represent the *r* value of Person’s correlation from light blue *r* < 0.69 to red *r* > 0.9. Full names of the abbreviations of the neuroanatomical structures are shown in Supplementary Table 1.

The isocortex extends topographically organized projections to the subpallial nuclei of the classic basal ganglia as well as to other neuroanatomical structures in the subpallium (Berendse et al., 1992; Reep et al., 2003; Oh et al., 2014; Chon et al., 2019). Projections from the isocortex to the subpallium integrate neural circuits that underlie a variety of functions including motor control and learning (Yin et al., 2004, 2005). To examine whether similar cadherin expression patterns exist between specific areas and layers of the isocortex and the nuclei of the subpallium, a Person’s correlation analysis of the mRNA expression of classical cadherins was conducted between each of the six layers of the nine areas of the isocortex and the fifty nuclei of the subpallium in the young adult P56 mouse CNS (ontological levels 11 and 12). The heatmap shown in Figure 9 displays the Person’s correlation values between the isocortex and the subpallium (Person’s correlation values and their statistical significance are shown in Supplementary Table 5). Distinct levels of correlation were observed between different cortical areas and the subpallium (Person’s correlation *r* values < 0.69 are here considered as low correlation, *r* values between 0.7 and 0.89 are considered as intermediate correlation, while *r* values > 0.9 are considered as high correlation).

Layer 2 of the frontal cortex shows moderate correlation with almost all subpallial structures, while layer 6 shows high correlation specifically with the olfactory tuberculum of the pallidum and striatum. Layers 5 and 6 of the parietal cortex moderately correlate with all parts of the subpallium, and the correlation is particularly high with the subpallial amygdala. In contrast, the intermediate layers 2–5 of the occipital cortex moderately correlate with the subpallial septum, paraseptal and central subpallium, but they display low correlation values with the subpallial amygdala. The insular cortex shows low to moderate correlation across the subpallium with no consistent preference for a particular layer. Layers 1 and 2 of the cingulate cortex are highly correlated with almost all structures of the subpallium, with the exception of the olfactory tuberculum of the paraseptal subpallium and the adjacent Calleja’s island of the central subpallium. The high correlation between upper cortical layers 1 and 2, and the subpallium is unique of the cingulate cortex. Layers 5 and 6 of the peri-ectorhinal cortex show moderate to high correlation across the subpallium, which is more pronounced between layer 5 and the subpallial amygdala. The retrosplenial and entorhinal cortex shows widespread moderate to high correlation across all layers and the correlation is consistently high between layer 3 and most of the subpallium. This analysis shows that the higher correlation of cadherin expression between the isocortex and the subpallial nuclei is between certain areas and layers of the cortex and specific nuclei of the subpallium.

## Discussion

Initial studies on tissue morphogenesis showed that dissociated embryonic cells regroup into organized tissue masses in which cells from highly cohesive tissues sort out from the cells belonging to less cohesive tissues, indicating that cell aggregation is regulated by characteristics of the cell surface that confer distinct cellular affinities and adhesive properties (Steinberg, 1963). These studies led to postulate the differential cell adhesion hypothesis of tissue morphogenesis, which states that specific cell sorting and tissue separation result from differences in cell adhesion (Foty and Steinberg, 2013). Since their discovery, classical cadherins have been implicated in the molecular mechanisms underlining differential cell adhesion (Takeichi et al., 1981; Hatta et al., 1988; Steinberg and Takeichi, 1994; Takeichi, 2018). The importance of cadherin-mediated adhesion in the formation of neural tissue was underscored by studies in which blockade of cadherin binding and ectopic cadherin expression disrupted tissue architecture (Matsunaga et al., 1988; Detrick et al., 1990; Fujimori et al., 1990; Bronner-Fraser et al., 1992).

The CNS is built by a complex developmental program that includes segmentation, nuclear organization, lamination, and formation of precise connections between distantly located neurons that configure an intricate array of neuronal networks. These morphogenetic events require specific cell sorting, generation of tissue boundaries, separation of cells into pools, and cellular stratification, which can be in part explained at the molecular level by differences in intercellular adhesion mediated by cadherins distinctive binding affinities and expression levels (Tepass et al., 2000; Foty and Steinberg, 2005; Katsamba et al., 2009; Fagotto, 2014). Examples of the role of cadherins in morphogenetic processes include the formation of a transverse tissue boundary that separates the pallium from the subpallium (Inoue et al., 2001), the role of a combinatorial expression of type II cadherins in the specific sorting of motor neurons into discrete pools in the spinal cord (Price et al., 2002; Bello et al., 2012), and the neuromeric segmentation of the neural tube (Hiraga et al., 2020). In addition, the ultrastructural similarities between cadherin-mediated adherens junctions and the synapse suggest that cadherins participate in the identification of neuronal partners and in the consolidation of the synaptic contact (Fannon and Colman, 1996; Uchida et al., 1996; Brusés, 2000). Indeed, several classical cadherins are expressed in a variety of synapses and contribute to pre and postsynaptic differentiation (Huntley and Benson, 1999; Rubio et al., 2005; Takeichi, 2007; Bekirov et al., 2008; Williams et al., 2011; Flannery and Brusés, 2012; Kuwako et al., 2014; Sanes and Zipursky, 2020). Cadherin-mediated adherens junction formed between radial glial cells are also necessary for generating and maintaining cell polarity required for the histological organization of the cerebral cortex (Hatakeyama et al., 2014; Veeraval et al., 2020).

The present study analyzed cadherins gene expression levels throughout the developing mouse CNS using publicly available ISH experiments conducted at the Allen Institute for Brain Science (Lein et al., 2007) to examine whether differential cadherin expression is associated with distinct morphogenetic events, including segmentation of the neural tube, formation of brain nuclei, and correlation of cadherin expression profiles between the cerebral cortex and the nuclei of the basal ganglia. During early CNS development, the neural tube undergoes a process of transverse segmentation along the anterior–posterior axis that results in the transient formation of morphogenetic units, and it is longitudinally divided along the dorsal–ventral axis into four plates (Puelles and Rubenstein, 1993, 2003; Martinez et al., 2012; Puelles et al., 2013). The segregation of cells into transverse segments of the neural tube is attributed to differential expression of cadherins (Wizenmann and Lumsden, 1997; Hiraga et al., 2020). For instance, *Cdh6* is transiently expressed along the hindbrain delineating an anterior boundary between rhombomeres r4 and r5, and it is later concentrated at rhombomere r6 (Inoue et al., 1997, 2009; Hiraga et al., 2020). The present study of cadherin-mediated relative adhesion based on mRNA expression levels and cadherin binding affinity coincides and expands previous findings, in that differential levels of cell adhesion mediated by *Cdh2*, *Cdh6*, *Cdh8*, and *Cdh11* are observed in distinct segments of the neural tube along the anterior–posterior axis. Based on the difference in cadherin expression levels required for specific cell sorting (Foty and Steinberg, 2005), the observed differences in cadherin-mediated relative adhesion between neural segments may be sufficient to separate cell groups. For instance, a difference of 2.4 times in the number of *Cdh2* molecules is sufficient to segregate cells into a peripheral and central cell mass *in vitro* (Foty and Steinberg, 2005). Based on the binding affinity of *Cdh2*, a 2.4 times difference in the number of molecules corresponds to a 3.8 times difference in relative adhesion. More than a four times difference in *Cdh2*, *Cdh6*, *Cdh9*, *Cdh8*, *Cdh11*, and *Cdh24* relative adhesion was observed between adjacent segments along the anterior–posterior axis. Although neuromeric segmentation occurs before E11.5 (the youngest stage here studied), the results suggest that the differences in relative adhesion along the neural tube promote the segregation and/or maintenance of cells within distinct segments. The narrow increase in *Cdh9* adhesion observed at the isthmus and rhombomere r1 points to a possible role of this cadherin in the tissue expansion associated with the formation of the cerebellum. The analysis of cadherin adhesion here presented only considers differences in relative adhesion mediated by a single cadherin subtype and does not include the impact that heterophilic cadherin binding and combinatorial expression of cadherins may have on cell adhesion and tissue organization. If these factors are considered, the actual differences in adhesive properties between segments of the CNS are expected to be much larger than the ones here reported.

The alar plate of the telencephalic vesicle is divided in pallium and subpallium. The subpallium is characterized by the proliferation and aggregation of cells forming brain nuclei including the classical basal ganglia (Puelles et al., 2000; García-López et al., 2008; Medina and Abellan, 2012). The cellular parcellation of the subpallium and the segregation of cells in patches and matrix in the caudo-putamen nucleus have been associated with the differential spatiotemporal expression of cell adhesion molecules (Hertel et al., 2008). The present analysis of the striatum and globus pallidus complexes (central subpallium) shows developmental variations in relative adhesion between the three strata, suggesting that differential cadherin-mediated adhesion contributes to the separation of subpallidal strata. Thereafter, each stratum gives rise to a distinct group of brain nuclei. The caudate originates from the periventricular stratum, the putamen, and the ventral striatum from the intermediate stratum, while the olfactory tuberculum originates from the superficial stratum. In the young adult CNS, the caudate and putamen display similar profiles of relative adhesion and the putamen relative adhesion differs from the one in the adjacent ventral striatum, which in turn differs from the relative adhesion of adjacent olfactory tuberculum. In the globus pallidus complex, the nuclei originated from the periventricular and intermediate strata display similar relative adhesion levels; in contrast, a pronounced difference is observed between the ventral and external pallium, which continues toward the nuclei derived from the superficial stratum (olfactory tuberculum). This analysis shows that differences in cadherin-mediated relative adhesion caused by both, different expression levels and cadherin subtype are associated with distinct neuronal groups during formation of the basal ganglia and in the adult mouse brain, suggesting that differential cadherin-mediated adhesion is involved in the formation of the brain nuclei.

The mouse cerebral cortex is histologically organized as a continuous laminated structure comprised of six cell layers (Kirkcaldie, 2012). The cortex is further divided into areas vinculated with their input-output connectivity and physiological role (Kirkcaldie, 2012). Classical cadherins expression in the cortex is cell-type specific, and different cortical areas and layers are enriched in distinct cadherin subtypes (Inoue and Sanes, 1997; Korematsu and Redies, 1997; Sanes and Yamagata, 1999; Krishna et al., 2009, 2011). The combinatorial expression pattern and differential binding affinities of classical cadherins contribute to the formation of precise neuronal connections by establishing homo and heterophilic adhesive bonds between apposed pre and postsynaptic membranes of specific neuronal partners (Poskanzer et al., 2003; Krishna et al., 2011; Osterhout et al., 2011; Duan et al., 2014, 2018; Basu et al., 2017; Sanes and Zipursky, 2020). The present study found that cadherin subtypes are similarly abundant in particular layers across cortical areas. *Cdh2* and *Cdh13* are expressed in an ascending gradient from layer 6 to the upper layers 2 and 3, while type II *Cdh8* and *Cdh11* are more abundant in the intermediate layers 2–5. In contrast, type II cadherins group A and B expression varies among cortical areas. This differential expression of classical cadherins in distinct cortical layers suggests that cadherin-mediated homo and heterophilic bonds drive formation of specific synaptic contacts between neurons from different cortical layers.

Distantly located neuroanatomical structures that are synaptically connected display matching cadherin expression patterns, suggesting that cadherin homophilic interactions drive the formation of specific connections between neurons (Redies and Takeichi, 1996; Suzuki et al., 1997). A detailed connectivity map between regions of the mouse brain generated by tracing axonal projections from defined cortical areas showed that the caudo-putamen, nucleus accumbens, and globus pallidus receive inputs from most cortical areas (Oh et al., 2014). Pearson’s correlation analysis conducted in the present study shows high correlation in cadherin expression profiles between certain cortical areas and subpallial nuclei. The level of correlation varies substantially among layers within the same cortical area, suggesting that differential and combinatorial cadherin expression may contribute to the formation of neural circuits between the pallium and the subpallium. Further analysis of cortical projections by axonal tracing from each cortical layer may uncover unidentified connections that are supported by cadherin binding.

The atypical *Cdh13* is linked to the cell membrane *via* a GPI moiety lacking the cytoskeletal interacting domain observed in classical cadherins (Ranscht and Dours-Zimmermann, 1991). The absence of a cytoplasmic domain precludes the regulation of cytoskeletal dynamics through the mechanisms observed in type I and type II cadherins. *Cdh13* homophilic binding affinity is comparable to the one observed between classical cadherins and supports calcium-dependent cell adhesion when heterologously expressed in cadherin deficient cells (Vestal and Ranscht, 1992; Ciatto et al., 2010; Brasch et al., 2018). *Cdh13* transdimerization is formed by a non-swapped interface that does not form *trans*-heterodimers with type I and type II cadherins (Ciatto et al., 2010). *Cdh13* is widely expressed throughout the developing CNS in a pattern that highly correlates with *Cdh2* neuroanatomical localization and expression levels. However, while *Cdh2* has neurite outgrowth promoting activity, *Cdh13* homophilic binding inhibits motor axon growth, suggesting that *Cdh13* may have a regulatory role on classical cadherins function (Sacristán et al., 1993; Ciatto et al., 2010). Outside the CNS, *Cdh13* expression is abundant in muscle and endothelial cells where it binds adiponectin and regulates lipid metabolism and angiogenesis (Hug et al., 2004), suggesting that *Cdh13* may regulate cell adhesion and neurodevelopmental processes by a still uncharacterized signaling mechanism.

In summary, the analysis of cadherin expression and relative adhesion throughout the developing mouse CNS shown here further supports previous evidence of the widespread role of classical cadherins in the formation of the nervous system and in the integration of neural networks. The inconsistent pattern and varied combinatorial expression of cadherins among neuroanatomical structures and developmental stages suggest that multiple cadherins are involved in each morphogenetic process and that their expression is regulated at a local level by distinct sets of transcription regulators. The large-scale systematic analysis of gene expression associated with the ontological brain anatomy conducted by the Allen Institute for Brain Science is instrumental for the detailed examination of the molecules involved in specific developmental events. The combination of gene expression data with the connectivity atlas and the electrophysiological analysis of neuronal responses may provide further evidence of the mechanisms whereby cadherins contribute to the formation and function of the CNS.

## Data Availability Statement

The original contributions presented in the study are included in the article/Supplementary Material. Further inquiries can be directed to the corresponding author/s.

## Author Contributions

JP, FR-V, and SDW retrieved data from databases, analyzed cadherin expression data, and generated tables and figures. ID conducted mathematical calculations of cadherin-mediated cell adhesion and Person’s correlation analysis, generated tables, and wrote the article. JLB conceived and directed the study, retrieved and analyzed the data, generated figures and tables, and wrote the article. All authors contributed to the article and approved the submitted version.

## Conflict of Interest

The authors declare that the research was conducted in the absence of any commercial or financial relationships that could be construed as a potential conflict of interest.

## Footnotes

1 https://alleninstitute.org/

2 http://developingmouse.brain-map.org

3 https://atlas.brain-map.org/

4 brain-map.org/api/index.html

5 http://api.brain-map.org/examples/rma_builder/rma_builder.html

6 https://portal.brain-map.org/atlases-and-data/rnaseq#Datasets

## Supplementary Material

Supplementary Figure 1 Representative images of tissue section from ISH experiments conducted at the Allen Brain Institute of E11.5 mouse embryos hybridized with probes for *Cdh2*, *Cdh6*, and *Cdh8*. The panels illustrate the varying expression pattern of each cadherin along the anterior–posterior axis. Arrowheads point to the areas of the neural tube in which each cadherin is detected. Method to retrieve ISH images used for the quantitative analysis is described in Section “Materials and Methods.” Images from the Allen Developing Mouse Brain Atlas: *Cdh2*, http://developingmouse.brain-map.org/experiment/show/100041183 (file 100041183_63); *Cdh6*, http://developingmouse.brain-map.org/experiment/show/100058753 (file 100058753_55); *Cdh11*, http://developingmouse.brain-map.org/experiment/show/100077809 (file 100077809_44). D, diencephalon; PH, pontine hindbrain; PPH, prepontine hindbrain; PMH, pontomedullary hindbrain; MH, medullary hindbrain.

Supplementary Figure 2 Comparison of cadherin mRNA expression energy (detected by ISH) between alar and basal plates along the anterior–posterior axis of the neural tube. The ratio of the expression energy of each cadherin between the alar and basal plate portion of a neuromeric segment was calculated to determine the fold difference in expression between plates. Values along the zero line represent the same mRNA expression energy in both plates, values above the zero line represent higher expression level in the alar plate, while values below the zero line represent higher expression in the basal plate (anterior is to the left). **(A–G)** Type I cadherins and *Cdh13*; **(A′–G′)** type II cadherins group A *Cdh6*, *Cdh9*, and *Cdh10*; **(A′′–G′′)** type II cadherins group B *Cdh7*, *Cdh12*, *Cdh18*, and *Cdh20*; **(A′′′–G′′′)** type II cadherins group C *Cdh8*, *Cdh11*, and *Cdh24*. **(A–A′′′)** E11.5; **(B–B′′′)** E13.5; **(C–C′′′)** E15.5; **(D–D′′′)** E18.5; **(E–E′′′)** P4; **(F–F′′′)** P14; **(G–G′′′)** P56. THy, terminal (rostral) hypothalamus; PHy, peduncular hypothalamus; p, prosomere; m, mesomere; is, isthmus; r, rhombomere.

Supplementary Figure 3 Representative images of the cerebral cortex of tissue sections from ISH experiments from P56 mouse hybridized with type I, type II and atypical *Cdh13* probes. The images illustrate the varying expression pattern of each cadherin in the different cortical layers. Method to retrieve ISH images used for the quantitative analysis is described in Section “Materials and Methods.” Scale bar, 200 μm. Images from the Allen Developing Mouse Brain Atlas: *Cdh2*, http://developingmouse.brain-map.org/experiment/show/79632275 *Cdh13*, http://developingmouse.brain-map.org/experiment/show/79360241 *Cdh6*, http://developingmouse.brain-map.org/experiment/show/100142546 *Cdh9*, http://developingmouse.brain-map.org/experiment/show/70719650 *Cdh10*, http://developingmouse.brain-map.org/experiment/show/75694362 *Cdh7*, http://developingmouse.brain-map.org/experiment/show/69540683 *Cdh12*, http://developingmouse.brain-map.org/experiment/show/73513627 *Cdh18*, http://developingmouse.brain-map.org/experiment/show/70611407 *Cdh20*, http://developingmouse.brain-map.org/experiment/show/77464878 *Cdh8*, http://developingmouse.brain-map.org/experiment/show/80525669 *Cdh11*, http://developingmouse.brain-map.org/experiment/show/77454677 *Cdh24*, http://developingmouse.brain-map.org/experiment/show/68862042

Supplementary Figure 4 Comparison of cadherins mRNA expression in the mouse cerebral cortex detected by ISH and scRNA-Seq. Classical cadherins and *Cdh13* mRNA expression energy detected by ISH in layers 1–6 of the P56 mouse frontal, parietal, temporal, and occipital cortical areas were normalized to the mRNA expression of *Actb* and the average of the normalized values for each cadherin was then calculated. mRNA levels detected by scRNA-Seq of each cadherin and *Actb* in ∼8-week-old mouse neocortex (1,093,785 total cells) were obtained from the scRNA-Seq database of the Allen Institute for Brain Science (https://portal.brain-map.org/atlases-and-data/rnaseq#Datasets). The Allen Mouse Transcriptomics Whole Cortex and Hippocampus 10X Genomics 2020 dataset was used for this analysis. The scRNA-Seq value of each cadherin in each of the 353 neuronal profiles was normalized to the scRNA-Seq value of *Actb*, and the average of the normalized values for each cadherin was calculated. The bar graph displays the average expression of each cadherin relative to *Cdh2* (*Cdh2* = 1). ISH, blue bars; scRNA-Seq, green bars. No statistically significant difference was detected between groups. Two-tailed *T*-test pairwise comparison *p* > 0.05.

Supplementary Table 1 Abbreviations of neuroanatomical structures of the dorsal pallium **(A),** subpallium **(B),** and the entire CNS **(C)** described in the Allen Reference Mouse Brain Atlas (https://atlas.brain-map.org/).

Supplementary Table 2 List of the section_data_set_id numbers corresponding to the experiments used in this study. The table indicates the ontology level up to which each developmental age has been annotated.

Supplementary Table 3 mRNA Expression energy values (obtained from the quantitative analysis of ISH experiments) of neuroanatomical structures (ontology level 3) from E11 to P56. Raw data of the heatmap shown in Figure 1.

Supplementary Table 4 mRNA Expression energy values (obtained from the quantitative analysis of ISH experiments) in each cerebral cortical area and layer in the P56 mouse CNS. Raw data of the heatmap displayed in Figure 8.

Supplementary Table 5 Person’s correlation *r* values and their statistical significance of the comparison of cadherin expression patterns between anatomical areas and layers of the isocortex and the nuclei of the subpallium shown in Figure 9 heatmap. ^∗^*p* < 0.05 and ^∗∗^*p* < 0.01.
