# Supplementary figures and images for: Differential Spatiotemporal Expression of Type I and Type II Cadherins Associated With the Segmentation of the Central Nervous System and Formation of Brain Nuclei in the Developing Mouse
Source: Front Mol Neurosci. 2021 Mar 23;14:633719. doi: 10.3389/fnmol.2021.633719 (PMC8021962; doi:10.3389/fnmol.2021.633719)

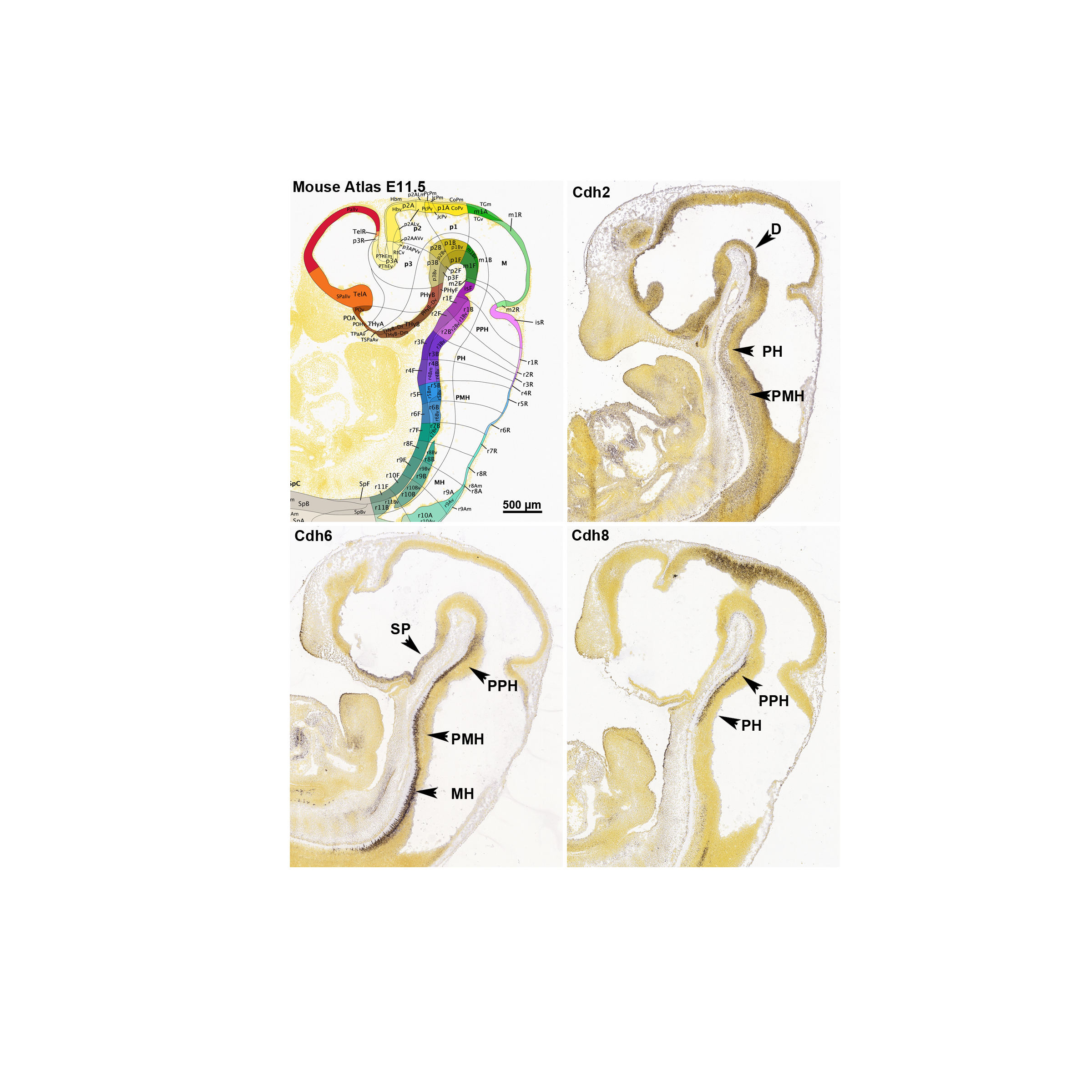

Supplement: Supplementary Figure 1 — Representative images of tissue section from ISH experiments conducted at the Allen Brain Institute of E11.5 mouse embryos hybridized with probes for Cdh2, Cdh6, and Cdh8. The panels illustrate the varying expression pattern of each cadherin along the anterior–posterior axis. Arrowheads point to the areas of the neural tube in which each cadherin is detected. Method to retrieve ISH images used for the quantitative analysis is described in Section “Materials and Methods.” Images from the Allen Developing Mouse Brain Atlas: Cdh2, http://developingmouse.brain-map.org/experiment/show/100041183 (file 100041183_63); Cdh6, http://developingmouse.brain-map.org/experiment/show/100058753 (file 100058753_55); Cdh11, http://developingmouse.brain-map.org/experiment/show/100077809 (file 100077809_44). D, diencephalon; PH, pontine hindbrain; PPH, prepontine hindbrain; PMH, pontomedullary hindbrain; MH, medullary hindbrain. [file Image_1.TIF]

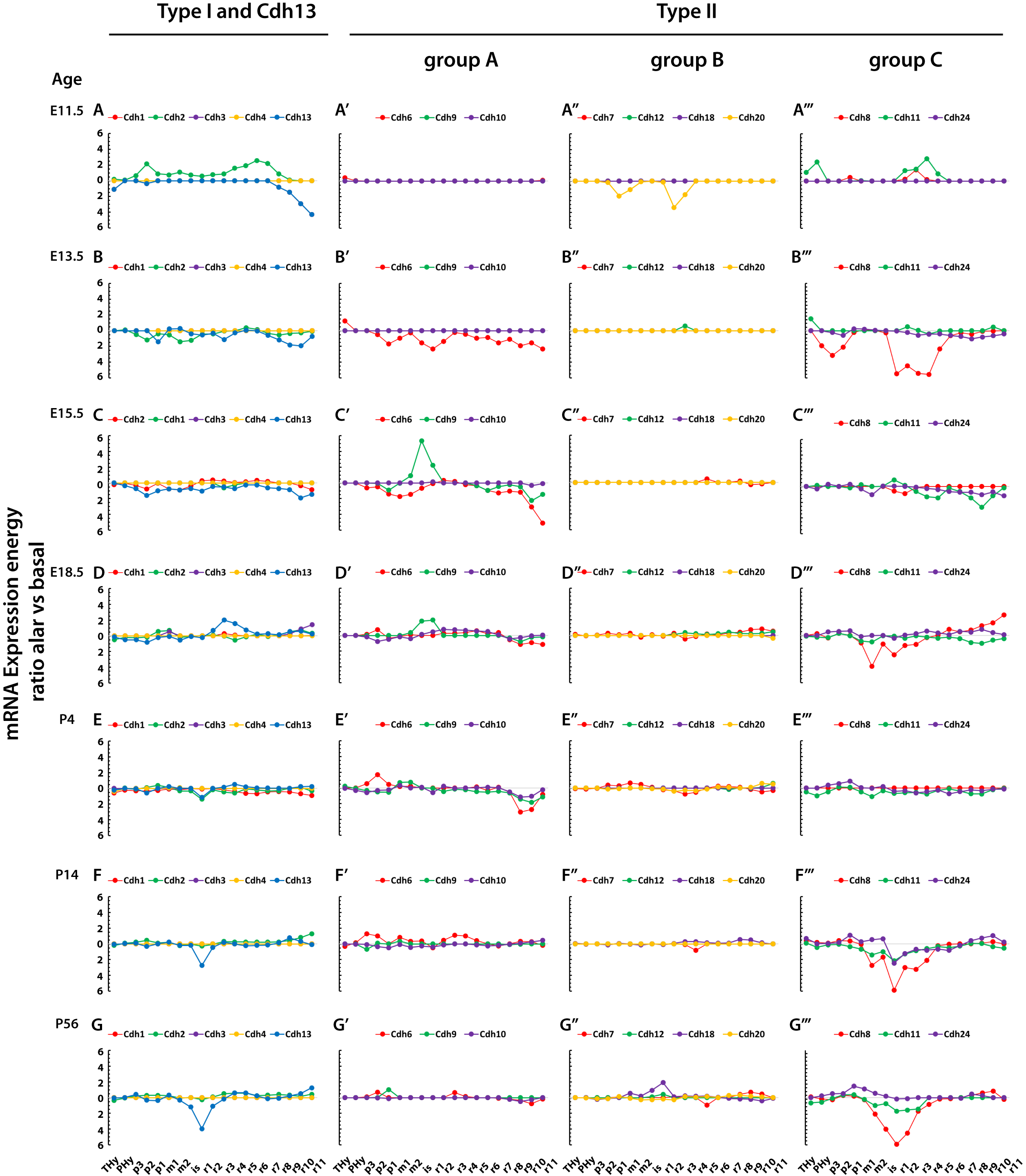

Supplement: Supplementary Figure 2 — Comparison of cadherin mRNA expression energy (detected by ISH) between alar and basal plates along the anterior–posterior axis of the neural tube. The ratio of the expression energy of each cadherin between the alar and basal plate portion of a neuromeric segment was calculated to determine the fold difference in expression between plates. Values along the zero line represent the same mRNA expression energy in both plates, values above the zero line represent higher expression level in the alar plate, while values below the zero line represent higher expression in the basal plate (anterior is to the left). (A–G) Type I cadherins and Cdh13; (A′–G′) type II cadherins group A Cdh6, Cdh9, and Cdh10; (A′′–G′′) type II cadherins group B Cdh7, Cdh12, Cdh18, and Cdh20; (A′′′–G′′′) type II cadherins group C Cdh8, Cdh11, and Cdh24. (A–A′′′) E11.5; (B–B′′′) E13.5; (C–C′′′) E15.5; (D–D′′′) E18.5; (E–E′′′) P4; (F–F′′′) P14; (G–G′′′) P56. THy, terminal (rostral) hypothalamus; PHy, peduncular hypothalamus; p, prosomere; m, mesomere; is, isthmus; r, rhombomere. [file Image_2.TIF]

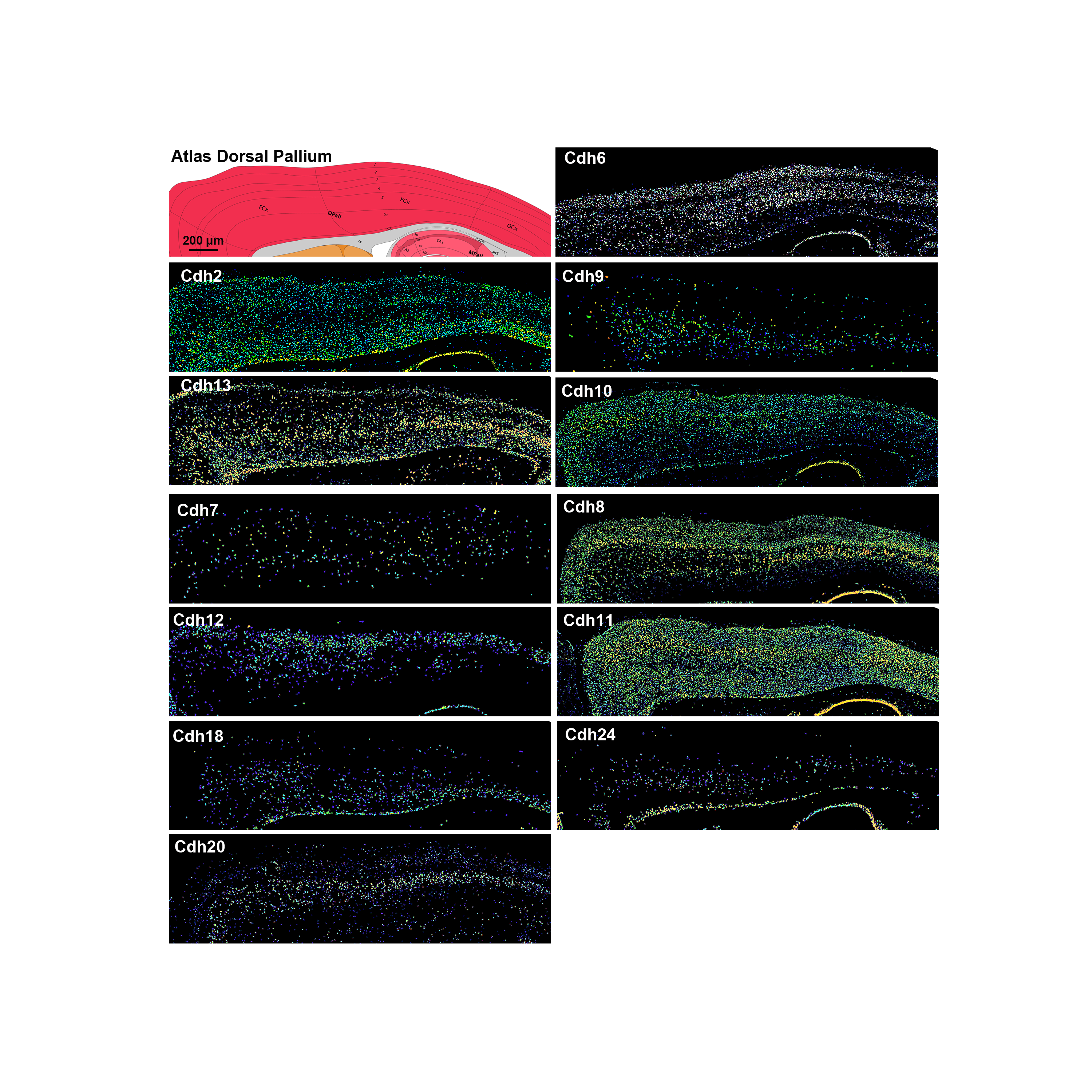

Supplement: Supplementary Figure 3 — Representative images of the cerebral cortex of tissue sections from ISH experiments from P56 mouse hybridized with type I, type II and atypical Cdh13 probes. The images illustrate the varying expression pattern of each cadherin in the different cortical layers. Method to retrieve ISH images used for the quantitative analysis is described in Section “Materials and Methods.” Scale bar, 200 μm. Images from the Allen Developing Mouse Brain Atlas: Cdh2, http://developingmouse.brain-map.org/experiment/show/79632275 Cdh13, http://developingmouse.brain-map.org/experiment/show/79360241 Cdh6, http://developingmouse.brain-map.org/experiment/show/100142546 Cdh9, http://developingmouse.brain-map.org/experiment/show/70719650 Cdh10, http://developingmouse.brain-map.org/experiment/show/75694362 Cdh7, http://developingmouse.brain-map.org/experiment/show/69540683 Cdh12, http://developingmouse.brain-map.org/experiment/show/73513627 Cdh18, http://developingmouse.brain-map.org/experiment/show/70611407 Cdh20, http://developingmouse.brain-map.org/experiment/show/77464878 Cdh8, http://developingmouse.brain-map.org/experiment/show/80525669 Cdh11, http://developingmouse.brain-map.org/experiment/show/77454677 Cdh24, http://developingmouse.brain-map.org/experiment/show/68862042 [file Image_3.TIF]

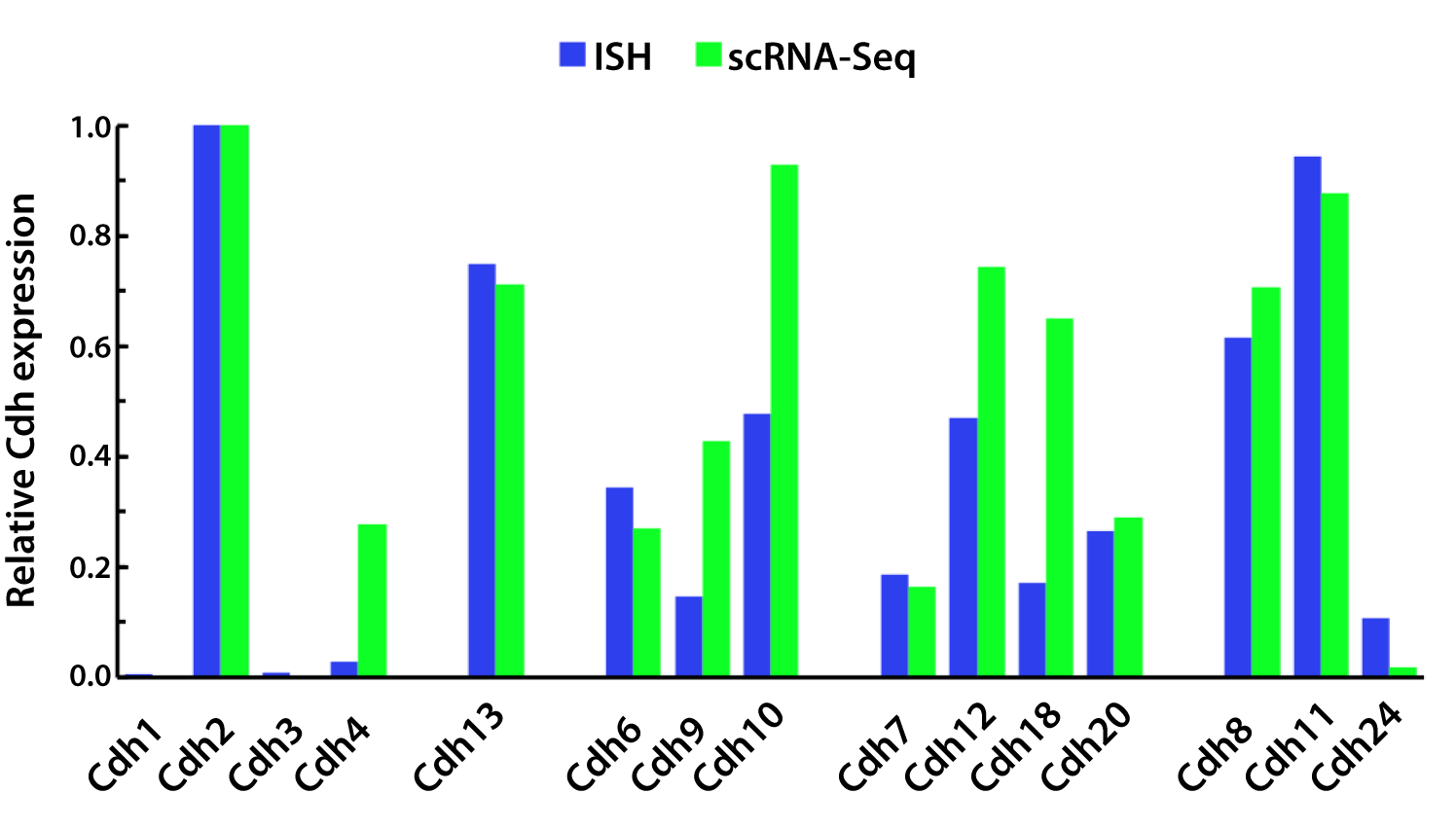

Supplement: Supplementary Figure 4 — Comparison of cadherins mRNA expression in the mouse cerebral cortex detected by ISH and scRNA-Seq. Classical cadherins and Cdh13 mRNA expression energy detected by ISH in layers 1–6 of the P56 mouse frontal, parietal, temporal, and occipital cortical areas were normalized to the mRNA expression of Actb and the average of the normalized values for each cadherin was then calculated. mRNA levels detected by scRNA-Seq of each cadherin and Actb in ∼8-week-old mouse neocortex (1,093,785 total cells) were obtained from the scRNA-Seq database of the Allen Institute for Brain Science (https://portal.brain-map.org/atlases-and-data/rnaseq#Datasets). The Allen Mouse Transcriptomics Whole Cortex and Hippocampus 10X Genomics 2020 dataset was used for this analysis. The scRNA-Seq value of each cadherin in each of the 353 neuronal profiles was normalized to the scRNA-Seq value of Actb, and the average of the normalized values for each cadherin was calculated. The bar graph displays the average expression of each cadherin relative to Cdh2 (Cdh2 = 1). ISH, blue bars; scRNA-Seq, green bars. No statistically significant difference was detected between groups. Two-tailed T-test pairwise comparison p > 0.05. [file Image_4.TIF]
